# Transcription coordinates histone amounts and genome content

**DOI:** 10.1038/s41467-021-24451-8

**Published:** 2021-07-09

**Authors:** Kora-Lee Claude, Daniela Bureik, Dimitra Chatzitheodoridou, Petia Adarska, Abhyudai Singh, Kurt M. Schmoller

**Affiliations:** 1 Institute of Functional Epigenetics, Helmholtz Zentrum München, Neuherberg, Germany; 2 Department of Electrical & Computer Engineering, University of Delaware, Newark, DE USA; 3 German Center for Diabetes Research (DZD), Neuherberg, Germany

**Keywords:** Cell biology, Transcription

## Abstract

Biochemical reactions typically depend on the concentrations of the molecules involved, and cell survival therefore critically depends on the concentration of proteins. To maintain constant protein concentrations during cell growth, global mRNA and protein synthesis rates are tightly linked to cell volume. While such regulation is appropriate for most proteins, certain cellular structures do not scale with cell volume. The most striking example of this is the genomic DNA, which doubles during the cell cycle and increases with ploidy, but is independent of cell volume. Here, we show that the amount of histone proteins is coupled to the DNA content, even though mRNA and protein synthesis globally increase with cell volume. As a consequence, and in contrast to the global trend, histone concentrations decrease with cell volume but increase with ploidy. We find that this distinct coordination of histone homeostasis and genome content is already achieved at the transcript level, and is an intrinsic property of histone promoters that does not require direct feedback mechanisms. Mathematical modeling and histone promoter truncations reveal a simple and generalizable mechanism to control the cell volume- and ploidy-dependence of a given gene through the balance of the initiation and elongation rates.

## Abstract (web summary)

Accurate regulation of protein concentrations according to changes in cell volume that accompany growth and changes in biosynthetic capacity is an important component of cellular homeostasis. Here, using the model organism *S. cerevisiae*, the authors show how histone production is quantitatively coupled to genome content through the intrinsic properties of histone promoters.

## Introduction

Maintaining accurate protein homeostasis despite cell growth and variability in cell volume is essential for cell function. Most proteins need to be kept at a constant, cell-volume-independent concentration. Since the amount of ribosomes and transcriptional machinery increases in proportion to cell volume, constant protein concentrations can be achieved through machinery-limited protein biogenesis, where protein synthesis depends on the availability of limiting machinery components and thus increases in direct proportion to cell volume^1,2^. While machinery-limited regulation can maintain constant concentrations of proteins, total mRNA, and individual transcripts^3–6^, it poses a conundrum for histones. As components of nucleosomes, histones are likely needed at a constant protein-to-DNA stoichiometry, implying that their amount should increase with ploidy but be independent of cell volume. In other words, histone concentration, i.e., amount per volume, should increase with ploidy but decrease with cell volume. Since accurate histone homeostasis is crucial for fundamental biological processes^7–10^ and to avoid toxic effects^11–13^, cells use several layers of regulation by translation, transcription and degradation to tightly coordinate histone production with genome replication^14–16^. However, how cells produce histones in proportion to genome content, even though protein biogenesis is generally linked to cell volume remains unclear.

Here, we use budding yeast as a model to show that histone protein amounts are coupled to genome content, resulting in a decrease of histone concentration in inverse proportion with cell volume, and an increase in direct proportion with ploidy. We find that this specific regulation of histones is achieved at the transcript level and does not necessarily require direct feedback mechanisms. While our data suggest that 3′-to-5′-degradation by the nuclear exosome is necessary for the correct decrease of concentration with cell volume, we show that histone promoters alone are sufficient to couple transcript amounts to gene copy number rather than cell volume. Our results suggest that this differential regulation of histones can be achieved through template-limited transcription, where mRNA synthesis is limited by the gene itself and does therefore not increase with cell volume. This provides a general mechanism by which cells can couple the amount of a subset of proteins to genome content while most protein concentrations are maintained constant.

## Results

### Histone protein concentrations decrease with cell volume and increase with ploidy

**Fig. 1:**
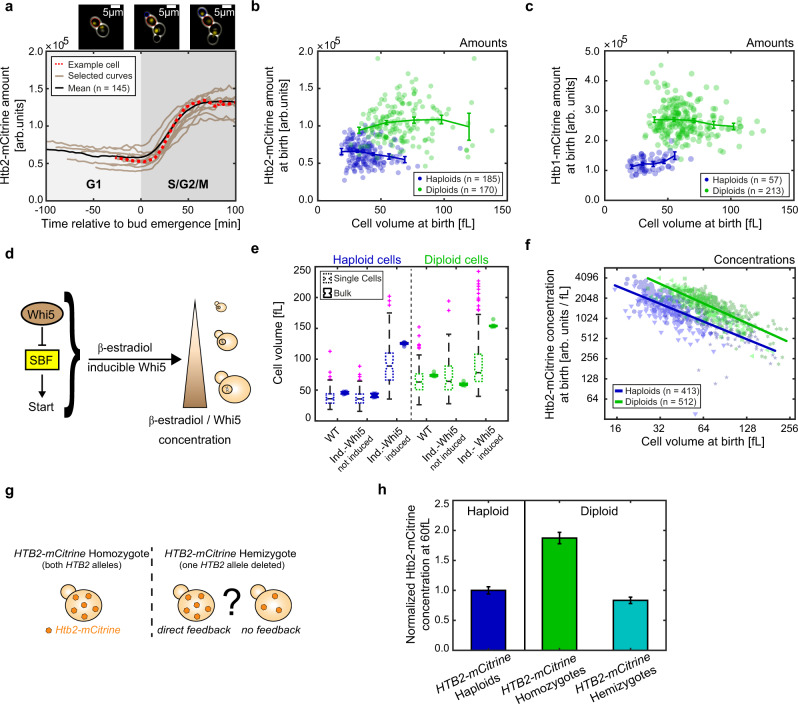
Htb1/2-mCitrine protein concentrations measured by live-cell fluorescence microscopy decrease with cell volume and increase with ploidy. **a** Htb2-mCitrine amounts during the first cell cycle of new-born cells. Red dashed trace highlights data corresponding to the outlined cell shown in the microscopy images (new-born cell: red outline, its bud: blue outline), brown traces show additional randomly selected example curves and the black line shows the mean of $n\,=\,145$ cells. All traces are aligned at the time of first bud emergence (*t* = 0). **b** Htb2-mCitrine amounts at birth for haploid (blue) and diploid (green) cells as a function of cell volume. Lines connect binned means with error bars indicating standard errors. **c** Htb1-mCitrine amounts at birth for haploid (blue) and diploid (green) cells as a function of cell volume. Lines connect binned means with error bars indicating standard errors. Note that the fluorescence intensities for Htb2 and Htb1 are not directly comparable due to differences in the microscopy settings. **d** Whi5 controls cell volume in a dose-dependent manner. To manipulate cell volume, *WHI5* is expressed from a β-estradiol-inducible promoter. Higher β-estradiol concentrations result in increased mean cell volumes. **e** Distribution of cell volumes for non-inducible (WT) and inducible haploids (blue) and diploids (green) measured at birth in *HTB2-mCitrine* single cells with live-cell fluorescence microscopy, or mean cell volumes in bulk populations of cells with untagged *HTB2* measured with a Coulter counter. Colored boxes highlight the 25- and 75-percentiles, whiskers extend to $\pm 2.7\sigma$ of the distributions and colored crosses highlight outliers. Black, horizontal lines indicate the median between single cells for single cell measurements (${n}_{{\rm{haploid}}}^{{\rm{WT}}}\,=\,185$, $\,{n}_{{\rm{haploid}}}^{{{{\rm{not}}\;{\rm{ind}}}}.}\,=\,120$,$\,{n}_{{\rm{haploid}}}^{{\rm{ind}}.}\,=\,108$, $\,{n}_{{\rm{diploid}}}^{{\rm{WT}}}\,=\,170$, $\,{n}_{{\rm{diploid}}}^{{{{\rm{not}}\;{\rm{ind}}}}.}\,=\,99$,$\,{n}_{{\rm{diploid}}}^{{\rm{ind}}}\,=\,243$) or the median of population means across seven biological replicates for bulk measurements. Notches indicate the 95% confidence interval of the median. Haploid cells were induced with 30 nM β-estradiol, diploid cells with 50 nM. **f** Htb2-mCitrine concentrations of non-inducible and inducible haploids and diploids as a function of cell volume are shown in a double-logarithmic plot. Individual data points for the different conditions are shown in blue for haploids (triangles for 0 nM, circles for WT, and stars for 30 nM) and green for diploids (triangles for 0 nM, squares for WT, and stars for 50 nM). Lines show linear fits to the double-logarithmic data, used to calculate the VDPs. **g** Illustration of the impact of potential feedback mechanisms on the concentration of Htb2-mCitrine concentration in a *HTB2-mCitrine/htb2Δ* hemizygous diploid compared to a *HTB2-mCitrine* homozygous diploid. **h** Htb2-mCitrine concentrations at 60 fL, estimated from the linear fit to the double-logarithmic dependence of concentration on cell volume, for haploids (blue bar, fit through $n\,=\,413$ cells), *HTB2-mCitrine* homozygous diploids (green bar, $n\,=\,512$ cells), and *HTB2-mCitrine/htb2Δ* hemizygous diploids (teal bar, $n\,=\,266$ cells), normalized on the concentration at 60 fL in haploids. Error bars are derived by error propagation of the 95% confidence interval of the linear fit at 60 fL.

Typically, total protein amounts as well as the amounts of individual types of protein increase roughly in direct proportion to cell volume to maintain constant concentrations. However, such regulation is inappropriate for histones, whose amount we predicted should be coupled to the cellular genome content instead. To test if this is the case, we chose the budding yeast histones *HTB1* and *HTB2*, the two genes encoding for the core histone H2B, as examples, because they can be fluorescently tagged without pronounced effects on cell growth. We endogenously tagged either *HTB1* or *HTB2* with the fluorescent protein *mCitrine* in a haploid strain, and measured cell volume and amount of Htb1/2-mCitrine over time in cycling cells by microfluidics-based live-cell fluorescence microscopy^17,18^. To obtain a large range of cell volumes, we grew cells on synthetic complete media with 2% glycerol 1% ethanol as a carbon source (SCGE). As expected^14^, we find that Htb1/2 amounts are constant during early G1, rapidly double during S-phase and reach a plateau before cytokinesis (Fig. 1a). We then quantified the Htb1/2-mCitrine amounts in new-born cells directly after cytokinesis and find that the amount of Htb1/2-mCitrine is largely constant, independent of cell volume (Fig. 1b, c). To further test whether histone amounts are coupled to genomic DNA content rather than cell volume, we next analyzed diploid strains in which both alleles of either *HTB1* or *HTB2* are tagged with *mCitrine*. Indeed, Htb1/2-mCitrine amounts in diploid cells are approximately a factor of two higher than in haploid cells (Fig. 1b, c). To more accurately compare Htb2 concentrations in haploids and diploids of similar volume, we sought to increase the overlapping range of observable volumes in both strains. For this purpose, we deleted the endogenous alleles of the G1/S inhibitor *WHI5* and integrated one copy of *WHI5* expressed from an artificial, β-estradiol-inducible promoter system^19^ (Fig. 1d). Using this system, we were able to increase the mean volume of steady-state exponentially growing populations by up to threefold through overexpression of Whi5 (Fig. 1e) without drastically affecting doubling times, budding indices or cell-cycle distributions (Supplementary Fig. 1). We repeated the microscopy experiments described above with the inducible-Whi5 haploid and diploid strains in the presence or absence of β-estradiol. Again, we find that Htb2-mCitrine amounts are only very weakly dependent on cell volume, but show a roughly twofold increase in diploid compared to haploid cells (Supplementary Fig. 2a). Consistently, we find that the concentration of Htb2-mCitrine at birth in both haploid and diploid cells decreases strongly with cell volume (Fig. 1f). To quantify this decrease, we performed a linear fit to the double-logarithmic data, and defined the slope as the volume-dependence-parameter (VDP). The observed VDPs of $-0.87\,\pm\, 0.04$ (haploids) and $-0.97\,\pm\, 0.03$ (diploids), respectively, are close to the value of −1 expected for proteins that are maintained at constant amount, resulting in a decrease of concentration with *c* ~ 1/*V*. In contrast, proteins that are maintained at constant concentration would show a VDP of 0.

In budding yeast, histones are known to be tightly regulated at several layers. In particular, some histone genes—including *HTB1*, but not *HTB2*—exhibit dosage compensation at the transcript level^20–22^. In addition, excess histones are known to be degraded^16^. In principle, a coupling of histone amounts to genomic DNA content could be achieved through such feedback mechanisms: For example, larger cells may produce histones in excess, and then degrade the surplus. Alternatively, direct feedback of histone protein amount on transcription could ensure that histones are expressed only until the protein amount matches the genome content. To test whether direct feedback of histone amounts on transcription, translation, or degradation is necessary to couple histone production to genome content, we again focused on Htb2, because it was already shown to not exhibit dosage compensation at the transcript level^21^. We constructed an inducible-Whi5 diploid strain in which we deleted one of the two *HTB2* alleles, while the other allele is tagged with *mCitrine* (Fig. 1g). If feedback were responsible for the coupling of Htb2 amount to genome content, the remaining *HTB2-mCitrine* allele should at least partially compensate for the deleted allele. However, consistent with the absence of any feedback, we find that Htb2-mCitrine concentrations are reduced by factor of two in the hemizygous compared to the homozygous diploid (Fig. 1h, Supplementary Fig. 2b). Moreover, at a characteristic volume of 60 fL, at which we find both haploid and diploid new-born cells, the concentration of Htb2-mCitrine in the hemizygous strain roughly equals the concentration in the haploid (Fig. 1h). While it seems likely that the reduced concentration of Htb2-mCitrine is compensated by an increased concentration of the other H2B, Htb1, our results suggest that no direct feedback is required to couple Htb2 amounts to genome content. Instead, Htb2 amounts are intrinsically determined by the *HTB2* gene copy number, independent of ploidy and cell volume.

### Histone mRNA concentrations decrease with cell volume

**Fig. 2:**
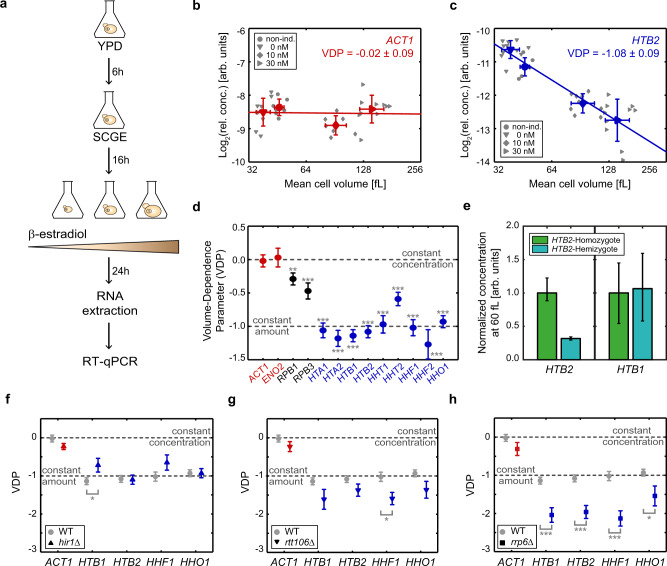
Histone mRNA concentrations decrease with cell volume and increase with gene copy number. **a** Experimental procedure for RT-qPCR measurements. Cells were grown for at least 24 h at the respective β-estradiol concentration before extracting total RNA and performing RT-qPCR. **b**, **c** Relative *ACT1* (**b**) or *HTB2* (**c**) mRNA concentrations (normalized on *RDN18*) for non-inducible and inducible haploid cells over mean cell volume are shown in a double-logarithmic plot. Individual data points for the different conditions (down-pointing triangles for 0 nM, circles for non-inducible, diamonds for 10 nM, right-pointing triangles for 30 nM) are shown in gray. Red (**b**) or blue (**c**) symbols indicate the mean of the different conditions with error bars indicating the standard deviations for ${n}_{{\rm{non}}-{\rm{ind}}.}^{{\rm{ACT}}1}\,=\,7, {n}_{0}^{{\rm{ACT}}1}\,=\,10, {n}_{10}^{{\rm{ACT}}1}\,=\,7, {{n}}_{30}^{{\rm{ACT}}1}\,=\,10$ (**b**), ${{n}}_{{\rm{non}}-{\rm{ind}}.}^{{\rm{HTB}}2}\,=\,7, {n}_{0}^{{\rm{HTB}}2}\,=\,11, {n}_{10}^{{\rm{HTB}}2}\,=\,9\,{\rm{and}}\, {n}_{30}^{{\rm{HTB}}2}\,=\,10$ (**c**) biological replicates. Lines show linear fits to the double-logarithmic data, with volume-dependence parameters (VDPs) determined as the slope of the fit. **d** Summary of the VDPs for all measured genes with error bars indicating the standard error (fit through ${n}_{{\rm{ACT}}1}\,=\,34$, ${n}_{{\rm{ENO}}2}\,=\,26$, ${n}_{{\rm{RPB}}1}=26$, ${n}_{{\rm{RPB}}3}=25$, ${n}_{{\rm{HTA}}1}=27$, ${n}_{{\rm{HTA}}2}=30$, ${n}_{{\rm{HTB}}1}=36$, ${n}_{{\rm{HTB}}2}=37$, ${n}_{{\rm{HHT}}1}=37$, ${n}_{{\rm{HHT}}2}=30$, ${n}_{{\rm{HHF}}1}=31$, ${n}_{{\rm{HHF}}2}=37$, and ${n}_{{\rm{HHO}}1}=37$ biological replicates); significance that the VDP is different from 0 was tested using linear regressions: **${p}_{{\rm{RPB}}1}=4.1\cdot {10}^{-3}$, ***${p}_{{\rm{RPB}}3}=9.0\cdot {10}^{-4}$, ***${p}_{{\rm{HTA}}1}=1.5\cdot {10}^{-9},$ ***${p}_{{\rm{HTA}}2}=1.8\cdot {10}^{-10}$, ***${p}_{{\rm{HTB}}1}=7.2\cdot {10}^{-14}$, ***${p}_{{\rm{HTB}}2}=8.0\cdot {10}^{-14}$, ***${p}_{{\rm{HHT}}1}=1.1\cdot {10}^{-8},$ ***$\,{p}_{{\rm{HHT}}2}=5.4\cdot {10}^{-6}$, ***$\,{p}_{{\rm{HHF}}1}=2.2\cdot {10}^{-9}$, ***$\,{p}_{{\rm{HHF}}2}=1.2\cdot {10}^{-6}$, ***$\,{p}_{{\rm{HHO}}1}=8.3\cdot {10}^{-13}$. **e** Median mRNA concentrations at 60 fL, estimated from the linear fit to the double-logarithmic dependence of concentration on cell volume, for *HTB2* (left) and *HTB1* (right) in diploid *HTB2* homozygous (green, fit through ${n}_{{\rm{HTB}}2}={n}_{{\rm{HTB}}1}=18$ biological replicates) and *HTB2/htb2*∆ hemizygous (teal, fit through ${n}_{{\rm{HTB}}2}={n}_{{\rm{HTB}}1}=18$ biological replicates) strains, normalized on the respective median concentration of the *HTB2*-homozygote. Error bars indicate the 2.5- and 97.5-percentiles around the median concentration ratio, determined from 10000 bootstrap samples. **f**–**h** Summary of VDPs for *hir1*∆ (**f**), *rtt106*∆ (**g**), as well as *rrp6*∆ (**h**) deletion strains. Error bars indicate the standard error of the VDPs (fit through ${n}_{{\rm{ACT}}1}^{{\rm{hir}}\triangle }=30$, ${n}_{{\rm{HTB}}1}^{{\rm{hir}}\triangle }=28$, ${n}_{{\rm{HTB}}2}^{{\rm{hir}}\triangle }=30$, ${n}_{{\rm{HHF}}1}^{{\rm{hir}}\triangle }=30$, ${n}_{{\rm{HHO}}1}^{{\rm{hir}}\triangle }=30$ (**f**), ${n}_{{\rm{ACT}}1}^{{\rm{rtt}}106\triangle }=12$, ${n}_{{\rm{HTB}}1}^{{\rm{rtt}}106\triangle }=12$, ${n}_{{\rm{HTB}}2}^{{\rm{rtt}}106\triangle }=12$, ${n}_{{\rm{HHF}}1}^{{\rm{rtt}}106\triangle }=12$, ${n}_{{\rm{HHO}}1}^{{\rm{rtt}}106\triangle }=11$ (**g**), ${n}_{{\rm{ACT}}1}^{{\rm{rrp}}6\triangle }=17$, ${n}_{{\rm{HTB}}1}^{{\rm{rrp}}6\triangle }=17$, ${n}_{{\rm{HTB}}2}^{{\rm{rrp}}6\triangle }=17$, ${n}_{{\rm{HHF}}1}^{{\rm{rrp}}6\triangle }=17$, ${n}_{{\rm{HHO}}1}^{{\rm{rrp}}\triangle }=17$(**h**) biological replicates). Significant VDP deviation from the wild-type VDP (carrying no deletion) was tested using linear regressions; *${p}_{{\rm{HTB}}1}^{{\rm{hir}}\triangle }=2.7\cdot {10}^{-2}$ (**f**), *${p}_{{\rm{HHF}}1}^{{\rm{rtt}}106\triangle }=2.3\cdot {10}^{-2}$ (**g**), ***${p}_{{\rm{HTB}}1}^{{\rm{rrp}}6\triangle }=6.8\cdot {10}^{-4}$, ***${p}_{{\rm{HTB}}2}^{{\rm{rrp}}6\triangle }=4.6\cdot {10}^{-4}$, ***${p}_{{\rm{HHF}}1}^{{\rm{rrp}}6\triangle }=2.6\cdot {10}^{-4}$, *${p}_{{\rm{HHO}}1}^{{\rm{rrp}}6\triangle }=2.0\cdot {10}^{-2}$ (**h**).

The fact that the decrease of histone Htb2 protein concentrations with cell volume is not simply a consequence of feedback, for example through excess protein degradation, suggests that it might already be established at the transcript level. To test if this is the case, we employed the Whi5-overexpression system to measure the cell-volume-dependence of transcript concentrations (Fig. 2a). Specifically, we grew wild-type haploid cells, as well as the inducible-Whi5 haploid cells at three different β-estradiol concentrations (0, 10, and 30 nM), on SCGE media, which led to a roughly fourfold range in mean cell volumes ranging from $39\pm 4$ fL to $143\pm 21$ fL (Supplementary Fig. 3a). To ensure steady-state conditions, we grew cells for at least 24 h at the respective β-estradiol concentration, before then measuring cell volume distribution, extracting total RNA, and performing reverse-transcription-qPCR (RT-qPCR). First, we measured the concentration of the ribosomal RNA *RDN18* relative to total RNA and found it to be constant (Supplementary Fig. 4a). This is consistent with the fact that ribosomal RNA constitutes the large majority of total RNA^23^, which itself is expected to increase in direct proportion to cell volume^24^, and allows us to now normalize other RT-qPCR measurements on *RDN18*.

Next, we quantified the mRNA concentrations of *ACT1* and *ENO2*, two genes that we expect to be expressed in proportion to cell volume such that the mRNA concentrations are maintained constant. Indeed, we find that the VDPs for both transcripts are not significantly different from 0 (Fig. 2b, d, Supplementary Fig. 4b). Interestingly, as previously suggested^25^ we observe a slight decrease in concentration for the transcripts of the RNA polymerase II subunits *RPB1* and *RPB3* with increasing cell volume (Fig. 2d, Supplementary Fig. 4c). We then quantified the concentrations of the transcripts of all core histone genes as well as the H1-like histone *HHO1*. In budding yeast, all core histone genes are present as two copies and expressed from bidirectional promoters controlling pairs of *H2A–H2B*^26^ or *H3–H4*^27^, respectively. Since the two copies of each core histone show high sequence similarity, we performed additional tests using deletion strains where possible to ensure qPCR primer specificity (Supplementary Table 2). We find that all histone transcripts show a significant decrease in concentration with cell volume, and mostly exhibit VDPs close to −1 (Fig. 2c, d, Supplementary Fig. 3b–d, Supplementary Fig. 4d). Thus, histone mRNA concentrations decrease with cell volume to ensure constant amounts—in contrast to global transcription, which increases with cell volume.

### Hir1-dependent feedback is not necessary for cell-volume-dependence of histone mRNA concentrations

The observation that histone transcript concentrations decrease with $c \sim 1/V$ suggests that, similar to histone protein amounts (Fig. 1h), also histone transcript amounts are determined by gene copy number. We therefore measured the concentrations of representative histone transcripts in inducible-Whi5 diploids homozygous or hemizygous for *HTB2*. Again, we find that all histones analyzed exhibit a VDP close to −1 (Supplementary Fig. 5a), and as observed for Htb2 protein concentrations (Fig. 1h), the concentration of *HTB2* transcripts at a characteristic volume of 60 fL is clearly reduced in hemizygous compared to homozygous diploids (Fig. 2e). We do not observe a significant overexpression of *HTB1* which is expected to compensate for the reduced *HTB2* transcript concentration^21^ (Fig. 2e). However, given that we see a roughly 50% overexpression of *HTB1* (and *HTA1*) upon deletion of *HTB2* in a haploid strain (Supplementary Fig. 5b), we note that we might not be able to resolve the comparably weaker (25%) overexpression of *HTB1* expected to compensate for one missing *HTB2* allele in the diploid strain given our experimental error.

So far, we have shown that in diploid cells with only one *HTB2* allele, the concentrations of *HTB2* transcript and protein are reduced compared to wild-type diploid cells. This highlights the absence of direct feedback mechanisms sensing and controlling the concentration of Htb2 with cell volume. However, extensive previous studies have shown that the eight budding yeast core histone genes show remarkably different modes of regulation. Specifically, only the gene pair *HTA1*-*HTB1* is known to exhibit dosage compensation, which is absent for *HTA2-HTB2*^20–22^. Moreover, three out of four core histone gene pairs, not including *HTA2-HTB2*, show negative feedback regulation of transcript concentration upon replication stress^14,28^. This feedback regulation is thought to be mediated by the HIR complex and to be dependent on *HIR1* and *RTT106*^29–31^. Thus, to test if HIR-dependent sensing and feedback regulation of histone transcript concentration may also be responsible for the cell-volume-dependence of HIR-regulated histone genes, we measured the cell-volume-dependence of representative histone genes (*HTB1*, *HTB2*, *HHF1*, and *HHO1*) in *hir1*∆ and *rtt106*∆ strains. While we observed a significantly weaker cell-volume-dependence for *HTB1* upon deletion of *HIR1*, we find that neither Hir1 nor Rtt106 are essential for a decrease of concentration with cell volume for any of the tested histone transcripts (Fig. 2f, g, Supplementary Fig. 5c, d).

### 3′-to-5′-degradation by the nuclear exosome is not necessary for cell-volume-dependence of histone mRNA concentrations

The fact that the correct dependence of histone transcript concentration on cell volume does not necessarily require direct feedback suggests that instead it is an intrinsic property of either transcription rate or mRNA degradation. To test if degradation from the 3′-end by the nuclear exosome is required, we analyzed the cell-volume-dependence of histone transcript concentrations in strains where we deleted *RRP6*, a component of the nuclear exosome exonuclease^32,33^. As shown in Fig. 2h, we find that also in *rrp6*∆ cells, histone transcript concentrations decrease with cell volume. Surprisingly, due to increased transcript concentrations in small cells (Supplementary Fig. 5e), this decrease with a VDP close to −2 is significantly stronger than in wild-type cells, suggesting that the volume-dependence of histone transcripts is modulated by Rrp6-dependent degradation. Thus, while degradation by the nuclear exosome is not needed for the volume-dependent decrease of histone transcript concentrations, it may contribute to achieve the correct VDP of −1.

### Histone promoters are sufficient for cell-volume-dependence of transcript concentrations

**Fig. 3:**
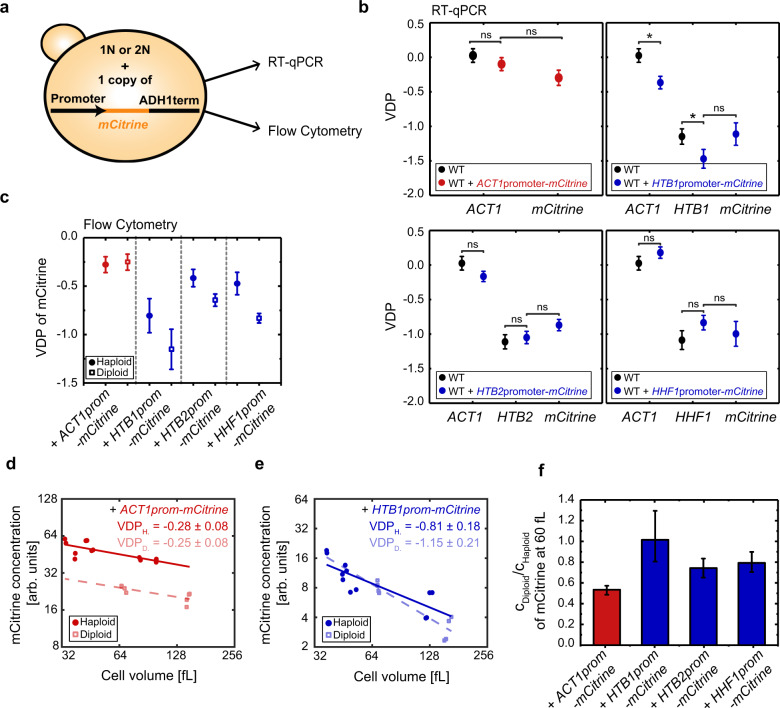
Histone promoters are sufficient for cell-volume- and ploidy-dependence of transcript concentrations. **a** Illustration of haploid (1N) or diploid (2N) strains carrying a single additional copy of a promoter of interest, driving the expression of the fluorescent reporter *mCitrine* regulated by the *ADH1* terminator. RT-qPCR or flow cytometry were used to analyze expression of the fluorescent reporter. **b** Summary of VDPs determined with RT-qPCR for the genes *ACT1*, *mCitrine*, and *HTB1*, *HTB2*, or *HHF1* for a haploid strain carrying an additional *ACT1* promoter (red circles), and haploid strains carrying an additional *HTB1*, *HTB2*, or *HHF1* promoter (blue circles) in comparison to a wild-type strain (black circles). VDPs were determined as the slope of the linear fit to the double-logarithmic dependence of concentration on cell volume (fit through ${n}_{{\rm{ACT}}1}^{{\rm{ACT}}1{\rm{prom}}}\,=\,36$, ${n}_{{\rm{mCitrine}}}^{{\rm{ACT}}1{\rm{prom}}}\,=\,36$, ${n}_{{\rm{ACT}}1}^{{\rm{HTB}}1{\rm{prom}}}\,=\,18$, ${n}_{{\rm{HTB}}1}^{{\rm{HTB}}1{\rm{prom}}}\,=\,17$, ${n}_{{\rm{mCitrine}}}^{{\rm{HTB}}1{\rm{prom}}}\,=\,18$, ${n}_{{\rm{ACT}}1}^{{\rm{HTB}}2{\rm{prom}}}\,=\,27$, ${n}_{{\rm{HTB}}2}^{{\rm{HTB}}2{\rm{prom}}}\,=\,27$, ${n}_{{\rm{mCitrine}}}^{{\rm{HTB}}2{\rm{prom}}}\,=\,27$, ${n}_{{\rm{ACT}}1}^{{\rm{HHF}}1{\rm{prom}}}\,=\,27$, ${n}_{{\rm{HHF}}1}^{{\rm{HHF}}1{\rm{prom}}}\,=\,27$, ${n}_{{\rm{mCitrine}}}^{{\rm{HHF}}1{\rm{prom}}}\,=\,27$ biological replicates), with error bars indicating the standard error of the VDPs. Significant VDP deviation between two genes was tested using linear regressions; *${p}_{{\rm{ACT}}1}^{{\rm{HTB}}1{\rm{prom}}}\,=\,1.0\cdot {10}^{-2}$, *${p}_{{\rm{HTB}}1}^{{\rm{HTB}}1{\rm{prom}}}\,=\,4.6\cdot {10}^{-2}$. **c** Summary of VDPs determined with flow cytometry for different strains in haploid (filled circles) and diploid (open squares) cells. VDPs were determined as the slope of the linear fit to the double-logarithmic dependence of concentration on cell volume (fit through ${n}_{{\rm{haploid}}}^{{\rm{ACT}}1{\rm{prom}}}\,=\,12$, ${n}_{{\rm{haploid}}}^{{\rm{HTB}}1{\rm{prom}}}=12$, ${n}_{{\rm{diploid}}}^{{\rm{HTB}}1{\rm{prom}}}=8$ , ${n}_{{\rm{haploid}}}^{{\rm{HTB}}2{\rm{prom}}}=12$, ${n}_{{\rm{diploid}}}^{{\rm{HTB}}2{\rm{prom}}}=8$, ${n}_{{\rm{haploid}}}^{{\rm{HHF}}1{\rm{prom}}}=12$, ${n}_{{\rm{diploid}}}^{{\rm{HHF}}1{\rm{prom}}}=8$ biological replicates), with error bars indicating the standard error of the VDPs. **d**, **e** *mCitrine* concentration, driven by an additional copy of the *ACT1* (**d**) or *HTB1* (**e**) promoter in haploid (filled circles) and diploid (open squares) cells, shown as a function of cell volume in a double-logarithmic plot. Lines show linear fits to the double-logarithmic data with volume-dependence parameters (VDPs) determined as the slope of the fit, with respective standard error. **f** Concentration of mCitrine, estimated from the linear fit to the double-logarithmic dependence of concentration on cell volume, in diploid cells compared to the concentration in haploid cells at 60 fL. Error bars indicate the 2.5- and 97.5-percentiles around the median concentration ratio, determined from 10,000 bootstrap samples.

Given that degradation from the 3′-end does not seem to be crucial for the cell-volume-dependent decrease of histone transcript concentration, we next asked whether the promoter alone is sufficient to establish this cell-volume-dependence. To address this, we created strains that carry additional copies of either the *ACT1* or a histone promoter (*HTB1*, *HTB2* or *HHF1*) driving expression of the fluorescent protein mCitrine, regulated by the identical *ADH1* terminator (Fig. 3a). We first confirmed that the additional promoter does not affect the VDPs of the endogenous histone and *ACT1* genes (Fig. 3b). Strikingly, we then find that the dependence of *mCitrine* transcript concentration on cell volume is determined by the promoter: If driven by the *ACT1* promoter, the VDP of *mCitrine* resembles that of endogenous *ACT1*; if driven by histone *HTB1*, *HTB2*, or *HHF1* promoter, it resembles that of the endogenous histone.

To verify this result using a different method, we made use of the fact that the fluorescent reporter mCitrine enables a fast experimental readout using flow cytometry (Fig. 3a). We analyzed the cell-volume-dependent fluorescence of mCitrine expressed from either the *ACT1* or a histone promoter (*HTB1*, *HTB2*, or *HHF1*). Consistent with the qPCR-based result, we find that all histone promoters tested show significantly negative VDPs in haploid and diploid cells, which confirms that flow cytometry can be used to qualitatively distinguish the distinct volume-dependences (Fig. 3c–e, Supplementary Fig. 6).

Histones not only need to be maintained at cell-volume-independent amounts, leading to a decrease of concentration with $1/V$, but also need to increase in proportion to cell ploidy (Fig. 1). This is in contrast to most other genes, which are maintained at a ploidy-independent concentration^34^. To test if the histone promoters are also sufficient to establish this distinct ploidy-dependence, we compared the expression level of the single *mCitrine* copy in diploid versus haploid cells. For *ACT1*, which needs to be maintained at a ploidy-independent concentration, we expect that a single gene allele in a diploid should produce half of the protein compared to a homozygous diploid or haploid of similar volume^2^. Indeed, for the *ACT1* promoter we find that at a given cell volume, the concentration of mCitrine expressed from a single additional promoter is 50% lower in diploids compared to haploids (Fig. 3d, f). In contrast, for each of the three histone promoters tested, we observe that the concentration in diploids is considerably higher than 50% of that in haploids of comparable volume, with a ratio close to 1 for the *HTB1* promoter (Fig. 3e, f, Supplementary Fig. 6). This demonstrates that in addition to setting the cell-volume-dependent decrease in concentration, regulation by the histone promoters also largely accounts for the fact that histones are needed in proportion to ploidy.

### Transcript concentrations during S-phase account for cell-volume-dependence of histone promoters

**Fig. 4:**
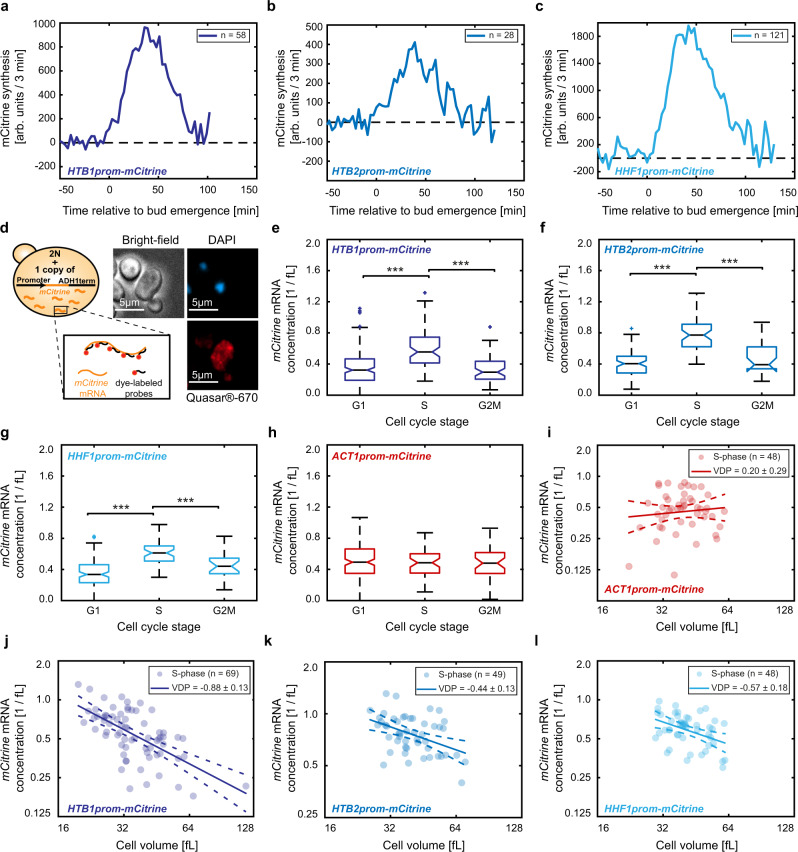
Cell-cycle-dependence does not account for the cell-volume-dependence of expression from histone promoters. **a**–**c** *mCitrine* synthesis rate measured by live-cell fluorescence microscopy during the first cell cycle of new-born diploid cells, when expressed from an additional *HTB1* (**a**), *HTB2* (**b**), or *HHF1* (**c**) promoter. Traces represent the mean of the moving averages over three frames of the single cell traces and are shown for the time span during which at least ten single cell traces were included in the average. All traces are aligned at the time of first bud emergence (*t* = 0). **d** Illustration of the smFISH experiments. Quasar^®^-670-labeled probes were used to count *mCitrine* mRNA spots in diploid cells carrying an additional promoter driving *mCitrine* expression. DAPI-staining of nuclear DNA and bright-field microscopy were used to classify cells as G1, S, or G2/M phase and to estimate cell volumes. Multiple images were taken per condition and at least two independent biological replicates were measured on different days. Example images show maximum intensity z-projections of diploid cells carrying an additional *HTB1* promoter; contrast was adjusted for visualization. **e**–**h** *mCitrine* mRNA concentration in G1-, S-, or G2/M-phases, estimated as the number of mRNA spots detected with smFISH in the whole cell including the bud and divided by the cell volume, for diploid cells expressing *mCitrine* from an additional *HTB1* (**e**), *HTB2* (**f**), *HHF1* (**g**), or *ACT1* (**h**) promoter. Colored boxes highlight the 25- and 75-percentiles, whiskers extend to $\pm 2.7\sigma$ of the distributions and colored crosses highlight outliers. Black, horizontal lines indicate the median between single cells for ${n}_{{\rm{G}}1}\,=\,158,{n}_{{\rm{S}}}\,=\,69,\ {n}_{{\rm{G}}2{\rm{M}}}\,=\,57$ (**e**), ${n}_{{\rm{G}}1}\,=\,77,\ {n}_{{\rm{S}}}\,=\,49,{n}_{{\rm{G}}2{\rm{M}}}\,=\,25$ (**f**), ${n}_{{\rm{G}}1}\,=\,113,{n}_{{\rm{S}}}\,=\,48,{n}_{{\rm{G}}2{\rm{M}}}\,=\,21$ (**g**), and ${n}_{{\rm{G}}1}\,=\,151,\ {n}_{{\rm{S}}}\,=\,48,\ {n}_{{\rm{G}}2{\rm{M}}}\,=\,38$ (**h**), with notches indicating the 95% confidence interval. Significances were tested using a two-tailed, two-sample t test at a confidence level *α* = 0.05, where applicable (between G1 and S-phase cells for (**f**), between all populations for (**g**, **h**)), or a Kruskal–Wallis test at a confidence level $\alpha \,=\,0.05$ otherwise; ***${p}_{{\rm{G}}1{\rm{vs}}\; {\rm{S}}}\,=\,1.0\cdot {10}^{-11},$ ***${p}_{{\rm{S}}\; {\rm{vs}}\; {\rm{G}}2{\rm{M}}}\,=\,1.2\cdot {10}^{-9}\,$(**e**), ***${p}_{{\rm{G}}1{\rm{vs}}\;{\rm{ S}}}\,=\,8.0\cdot {10}^{-21},$ ***${p}_{{\rm{S}}\; {\rm{vs}}\; {\rm{G}}2{\rm{M}}}\,=\,6.5\cdot {10}^{-7}\,$(**f**), ***${p}_{{\rm{G}}1{\rm{vs}}\; {\rm{S}}}\,=\,3.5\cdot {10}^{-16},$ ***${p}_{{\rm{S}}\; {\rm{vs}}\;{\rm{ G}}2{\rm{M}}}\,=\,5.4\cdot {10}^{-4}\,$ (**g**). A diploid strain carrying no *mCitrine* allele was used as a control to test that smFISH signal is specific (Supplementary Fig. 7h). **i**–**l** *mCitrine* mRNA concentration in S-phase cells, expressed from an additional *ACT1* (**i**) or *HTB1* (**j**), *HTB2* (**k**), or *HHF1* (**l**) promoter, shown as a function of cell volume in a double-logarithmic plot. Solid lines show linear fits to the double-logarithmic data, dashed lines represent the 95% confidence intervals of the fit. Volume-dependence parameters (VDPs) were determined as the slope of the fit, with respective standard error.

So far, we have shown that for *mCitrine* transcripts expressed from histone promoters, average concentrations in asynchronous populations decrease with average cell volume. It is well established that histone synthesis is temporally linked to DNA replication, restricting histone expression to late G1 and S-phase. While also protein and mRNA degradation contribute to this cell-cycle-dependence, regulation mediated by the promoter is sufficient for increased expression in S-phase^14,35,36^. Indeed, using live-cell microscopy we confirmed that all our histone promoter constructs exhibit a peak of mCitrine synthesis after bud emergence, roughly corresponding to S-phase (Fig. 4a–c). Thus, two possible scenarios could account for the decreased *mCitrine* transcript concentration in large compared to smaller cells. One explanation is that during the period in which *mCitrine* is expressed, the transcript concentration decreases in larger cells. However, another explanation would be that the *mCitrine* transcript concentration during S-phase is constant independent of cell volume, but S-phase duration decreases in large cells. In an asynchronous cell population, this would then lead to a decrease in the fraction of cells that express *mCitrine*, which in turn would result in a cell-volume-dependent decrease of the average transcript concentration.

In the live-cell microscopy experiments described above, we did not observe a relevant decrease in the mCitrine production period with cell volume (Supplementary Fig. 7a–c). To gain further insight into the potential contribution of a cell-volume-dependent cell-cycle-dependence, we performed single-molecule fluorescence in situ hybridization (smFISH), which allows us to count *mCitrine* mRNA molecules in single cells. At the same time, we can use bright-field microscopy to estimate cell volume and – together with a DAPI-stain to visualize the nucleus—classify cells as being in G1, S, or G2/M phase (Fig. 4d). Consistent with our live-cell microscopy result, we again observe an increased expression during S-phase for all histone promoters analyzed (Fig. 4e–g, Supplementary Fig. 7d–f). In contrast, the *ACT1* promoter results in more uniform expression throughout the cell cycle (Fig. 4h, Supplementary Fig. 7g). To test whether expression during S-phase accounts for the overall cell-volume-dependence, we analyzed the concentration of transcripts in S-phase as a function of cell volume. Strikingly, we find that all three histone promoters (*HTB1*, *HTB2*, and *HHF1* promoters) but not the *ACT1* promoter lead to a significant decrease of transcript concentration with cell volume (Fig. 4i–l).

### Different cell-volume and ploidy dependences can be explained by competition of promoters for limiting transcriptional machinery

**Fig. 5:**
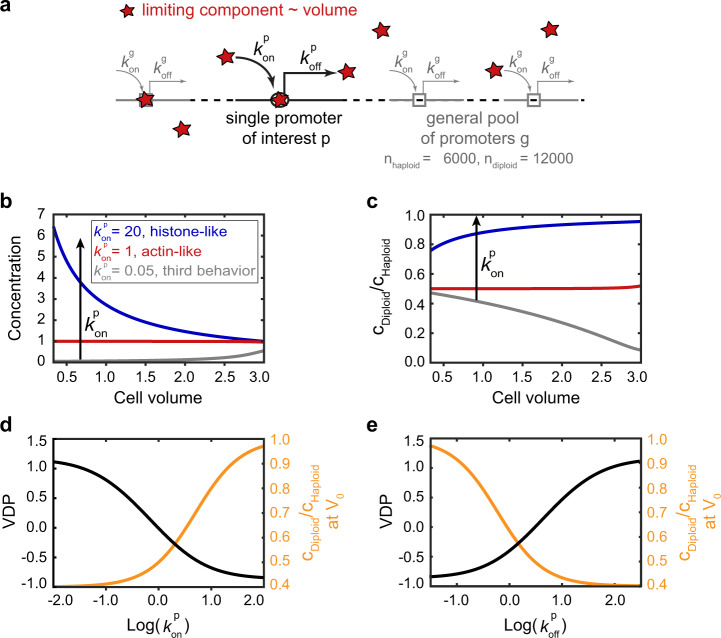
Minimal model for the dependence of transcription rate of one specific promoter of interest on cell volume and ploidy. **a** The model includes two classes of promoters: the general pool of promoters $g$ and the specific promoter of interest $p$ with their respective initiation rates ${k}_{{\rm{on}}}^{{\rm{p}}}\,$ or $\,{k}_{{\rm{on}}}^{g}$, describing the binding of the limiting machinery and off-rates ${k}_{{\rm{off}}}^{{\rm{p}}}\,$ or $\,{k}_{{\rm{off}}}^{g}$, summarizing all other steps of transcription. **b**–**e** The model predicts that tuning ${\,k}_{{\rm{on}}}^{{\rm{p}}}$ or ${{k}}_{{\rm{off}}}^{p}\,$ while keeping all other parameters fixed (${c}_{{\rm{TM}}}\,=\,2000,{k}_{{\rm{on}}}^{g}\,=\,1,{k}_{{\rm{off}}}^{g}\,=\,{k}_{{\rm{off}}}^{{\rm{p}}}\,=\,3,$ for tuning ${\,k}_{{\rm{on}}}^{{\rm{p}}},$ or ${c}_{{\rm{TM}}}\,=\,2000,{k}_{{\rm{off}}}^{g}\,=\,3,{k}_{{\rm{on}}}^{g}\,=\,{k}_{{\rm{on}}}^{{\rm{p}}}\,=\,1,$ for tuning ${\,k}_{{\rm{off}}}^{{\rm{p}}}$) results in a qualitative change of the cell volume-dependence of transcript concentration obtained from the specific promoter (**b**), as well as a change in the ratio between the concentration in diploid cells and the concentration in haploid cells (**c**). **d**, **e** Model prediction for the VDP (right, black) and for the ratio between the concentration in diploid cells and the concentration in haploid cells at a characteristic volume ${V}_{0}\,=\,1$ (left, orange) as a function of ${\,k}_{{\rm{on}}}^{{\rm{p}}}$ (**d**) or ${\,k}_{{\rm{off}}}^{{\rm{p}}}$ (**e**).

Taken together, our results suggest that while most genes are transcribed at a rate proportional to cell volume to maintain constant concentrations, transcripts controlled by histone promoters are instead transcribed at a cell-volume-independent rate to couple histone production to DNA content. To better understand how the transcription rate of one specific promoter depends on cell volume and ploidy context, we revisited a minimal model for transcription that was in essence proposed by Heldt et al.^37^ (Fig. 5a). We further simplified the model by not explicitly modeling the dynamics of cell growth. Instead, we assume that transcription can be described by a single (limiting) component of the transcriptional machinery, TM, whose amount increases in proportion to cell volume. In other words, its concentration ${c}_{\mathrm{TM}}$ stays constant. We then considered two classes of promoters, a specific promoter of interest, $p$, present as a single copy, and a general pool of promoters, $g$, which are present as ${n}_{{\mathrm{h}}}=6000$ in haploids or ${n}_{{\mathrm{d}}}=12000$ copies in diploids. Each promoter is competing for the transcriptional machinery, and is modeled as a single binding site for TM. Initiation, i.e., binding of the machinery, occurs at a rate ${k}_{\mathrm{on}}^{\mathrm{p}}$ or ${k}_{\mathrm{on}}^{g}$, respectively. Furthermore, we assume that all other steps of transcription can be summarized in a single rate-limiting step, occurring at a rate ${k}_{\mathrm{off}}^{\mathrm{p}}$ or ${k}_{\mathrm{off}}^{g}$, respectively. Each transcript is then degraded with the same rate$\,{k}_{{\mathrm{deg }}}=1$. Depending on the parameters chosen for the specific promoter, the model predicts qualitatively different dependences of transcript concentration on cell-volume and ploidy (Fig. 5b–e)^37^.

For example, at a given ${{k}}_{{\mathrm{off}}}^{{\mathrm{p}}}$, a high on-rate ${k}_{{\mathrm{on}}}^{{\mathrm{p}}}$ (${k}_{{\mathrm{on}}}^{{\mathrm{p}}}\gg {k}_{\mathrm{on}}^{g}$) can result in histone-promoter-like behavior, i.e., cell volume-dependent but ploidy-independent transcript concentration. This can be understood considering that due to the higher on-rate compared to the general pool of promoters, the promoter of interest is already saturated with transcriptional machinery at very small cell volumes. A further increase of the available machinery at larger cell volumes does therefore not result in a higher occupancy with transcriptional machinery, leading to a constant, cell-volume-independent transcription rate. Thus, the transcript concentration obtained from a single promoter of interest is independent of ploidy but decreases with cell volume. A homozygous diploid carrying two of the promoters of interest will therefore show a twofold higher concentration than a haploid with the same volume. In contrast, at lower ${k}_{{\mathrm{on}}}^{{\mathrm{p}}}$ (${k}_{{\mathrm{on}}}^{{\mathrm{p}}}\approx {k}_{\mathrm{on}}^{g}$) we observe actin-promoter-like behavior, i.e., cell volume-independent but ploidy-dependent transcript concentration. In this regime, both the specific and the general promoters compete equally for the transcriptional machinery. As long as not all promoters are saturated, transcription rate therefore increases with the amount of available transcriptional machinery, and thus cell volume. In diploid cells, the single promoter of interest competes with twice the number of general promoters, leading to a roughly twofold reduction of the transcription rate compared to haploids with equal cell volume.

Interestingly, at very low ${k}_{{\mathrm{on}}}^{{\mathrm{p}}}$ (${k}_{{\mathrm{on}}}^{{\mathrm{p}}}\ll {k}_{\mathrm{on}}^{g}$) we observe a third type of behavior, in which transcript concentration increases with cell volume. Because the affinity of the promoter of interest to the transcriptional machinery is very low compared to that of the general promoters, the occupancy is very low as long as the general promoters are not saturated. Only once the general promoters approach saturation, machinery becomes available for the promoter of interest, then leading to a nonlinear increase of transcription rate with cell volume.

### Histone-promoter truncations lead to a switch from histone-like to actin-like behavior

**Fig. 6:**
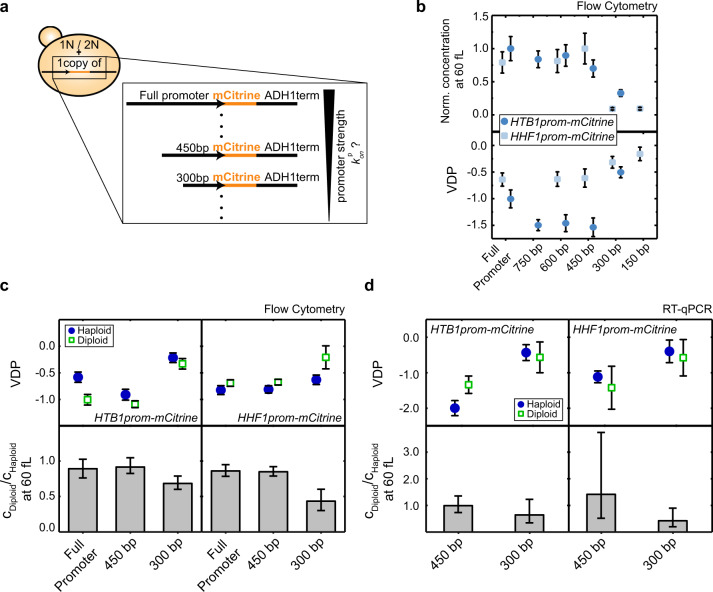
Reducing the strength of a histone promoter shifts its behavior from histone-like to actin-like. **a** Illustration of a series of haploid and diploid strains carrying a single additional copy of increasingly shorter fragments of promoters driving *mCitrine* expression, each truncated from the 5′-end. **b** mCitrine concentration at 60 fL normalized on maximum concentration of the respective promoter (upper panel) and VDP of mCitrine (bottom panel) determined by flow cytometry for the respective promoter truncations of the *HTB1* promoter (dark blue circles) and the *HHF1* promoter (light blue squares) driving *mCitrine* expression, integrated in haploid cells. Concentrations were estimated from a linear fit to the double logarithmic dependence of concentration on cell volume, VDPs were determined as the slope of the linear fit (fit through$\,{n}_{{\rm{full}}}^{{\rm{HTB}}1{\rm{prom}}}\,=\,12$,$\,{n}_{750{\rm{bp}}}^{{\rm{HTB}}1{\rm{prom}}}\,=\,15$,$\,{n}_{600{\rm{bp}}}^{{\rm{HTB}}1{\rm{prom}}}\,=\,15$,$\,{n}_{450{\rm{bp}}}^{{\rm{HTB}}1{\rm{prom}}}\,=\,15$,$\,{n}_{300{\rm{bp}}}^{{\rm{HTB}}1{\rm{prom}}}\,=\,15$, and $\,{n}_{{\rm{full}}}^{{\rm{HHF}}1{\rm{prom}}}\,=\,12$, $\,{n}_{600{\rm{bp}}}^{{\rm{HHF}}1{\rm{prom}}}\,=\,15$,$\,{n}_{450{\rm{bp}}}^{{\rm{HHF}}1{\rm{prom}}}\,=\,15$,$\,{n}_{300{\rm{bp}}}^{{\rm{HHF}}1{\rm{prom}}}\,=\,15$,$\,{n}_{150{\rm{bp}}}^{{\rm{HHF}}1{\rm{prom}}}\,=\,15$ biological replicates). Error bars in the upper panel are derived by error propagation of the 95% confidence interval of the linear fit at 60 fL. In the bottom panel, error bars show the standard error of the VDPs. **c** VDP of mCitrine in haploid (blue filled circles) and diploid (green open squares) cells (upper panel) and mCitrine concentration at 60 fL in diploids compared to the concentration in haploids (bottom panel) determined by flow cytometry. Left shows results for the *HTB1* promoter truncations, right shows results for the *HHF1* promoter truncations. Concentrations were estimated from a linear fit to the double logarithmic dependence of concentration on cell volume, VDPs were determined as the slope of the linear fit (fit through $\,{n}_{{\rm{full}},{\rm{haploid}}}^{{\rm{HTB}}1{\rm{prom}}}\,=\,27$, ${n}_{{\rm{full}},{\rm{diploid}}}^{{\rm{HTB}}1{\rm{prom}}}\,=\,18$, ${n}_{450{\rm{bp}},{\rm{haploid}}}^{{\rm{HTB}}1{\rm{prom}}}\,=\,27$, ${n}_{450{\rm{bp}},{\rm{diploid}}}^{{\rm{HTB}}1{\rm{prom}}}\,=\,27$, ${n}_{300{\rm{bp}},{\rm{haploid}}}^{{\rm{HTB}}1{\rm{prom}}}\,=\,27$, ${n}_{300{\rm{bp}},{\rm{diploid}}}^{{\rm{HTB}}1{\rm{prom}}}\,=\,27$, and ${n}_{{\rm{full}},{\rm{haploid}}}^{{\rm{HHF}}1{\rm{prom}}}\,=\,27$, ${n}_{{\rm{full}},{\rm{diploid}}}^{{\rm{HHF}}1{\rm{prom}}}\,=\,18$, ${n}_{450{\rm{bp}},{\rm{haploid}}}^{{\rm{HHF}}1{\rm{prom}}}\,=\,27$, ${n}_{450{\rm{bp}},{\rm{diploid}}}^{{\rm{HHF}}1{\rm{prom}}}\,=\,18$, ${n}_{300{\rm{bp}},{\rm{haploid}}}^{{\rm{HHF}}1{\rm{prom}}}\,=\,27$, ${n}_{300{\rm{bp}},{\rm{diploid}}}^{{\rm{HHF}}1{\rm{prom}}}\,=\,17$ biological replicates). Error bars in the upper panels show the standard error of the VDPs. In the bottom panel, error bars indicate the 2.5- and 97.5-percentiles around the median concentration ratio, determined from 10,000 bootstrap samples. **d** VDP of *mCitrine* in haploid (blue filled circles) and diploid (green open squares) cells (upper panel) and *mCitrine* mRNA concentration at 60 fL in diploids compared to the concentration in haploids (bottom panel) determined by RT-qPCR for *HTB1* and *HHF1* promoter truncations driving *mCitrine* expression. Concentrations were estimated from a linear fit to the double logarithmic dependence of concentration on cell volume, VDPs were determined as the slope of the linear fit (fit through ${{n}}_{450{\rm{bp}},{\rm{haploid}}}^{{\rm{HTB}}1{\rm{prom}}}\,=\,15$, ${n}_{450{\rm{bp}},{\rm{diploid}}}^{{\rm{HTB}}1{\rm{prom}}}\,=\,14$, ${n}_{300{\rm{bp}},{\rm{haploid}}}^{{\rm{HTB}}1{\rm{prom}}}\,=\,16$, ${n}_{300{\rm{bp}},{\rm{diploid}}}^{{\rm{HTB}}1{\rm{prom}}}\,=\,12$, and, ${n}_{450{\rm{bp}},{\rm{haploid}}}^{{\rm{HHF}}1{\rm{prom}}}\,=\,12$, ${n}_{450{\rm{bp}},{\rm{diploid}}}^{{\rm{HHF}}1{\rm{prom}}}\,=\,12$, ${n}_{300{\rm{bp}},{\rm{haploid}}}^{{\rm{HHF}}1{\rm{prom}}}\,=\,11$, ${n}_{300{\rm{bp}},{\rm{diploid}}}^{{\rm{HHF}}1{\rm{prom}}}\,=\,12$ biological replicates). Error bars in the upper panel show the standard error. Error bars in the bottom panel indicate the 2.5- and 97.5-percentiles around the median concentration ratio, determined from 10,000 bootstrap samples.

One key prediction of this model is that if all other parameters are fixed, reducing ${k}_{{\mathrm{on}}}^{{\mathrm{p}}}$ for a histone-like promoter should eventually shift its behavior to that of an actin-like promoter (Fig. 5d). To experimentally test this prediction, we aimed to decrease the initiation rate ${k}_{{\mathrm{on}}}^{{\mathrm{p}}}$ of the *HHF1* and *HTB1* promoters by creating series of haploid and diploid strains with increasingly shorter fragments of the promoters, each truncated from the 5′-end (Fig. 6a). Again, we used flow cytometry to analyze mCitrine expression driven by these additional, endogenously integrated promoter fragments. For both promoters we observe a decrease of mCitrine expression once part of the known upstream activating sequences (UASs)^35^ are truncated (Fig. 6b, Supplementary Fig. 8a). Fully consistent with the model, for both promoters, and for haploids and diploids, this drop in expression coincides with a change of the VDP toward $0$ (Fig. 6b, c, Supplementary Fig. 8b, c). At the same time and also consistent with the model, the ratio of the mCitrine concentration at a given volume in diploid compared to haploid cells decreases from close to 1 toward $0.5$ (Fig. 6c). Thus, our analysis shows that for both the *HHF1* and *HTB1* promoter truncation series, a transition from histone-like to actin-like behavior occurs between the 450 and 300 bp truncations.

While we consistently observe the same qualitative trend in flow cytometry measurements, we found that the exact VDP measured with flow cytometry depended on the flow cytometry settings, which need to be adjusted depending on the observed cell-volume range. Thus, to quantitatively confirm our results, we repeated the experiment for the 450 and 300 bp truncations of the *HTB1* and *HHF1* promoters using RT-qPCR. Again, we observe a change in the VDP toward 0, and a decrease of the ratio of the mCitrine concentration between diploid and haploid cells from close to 1 to close to 0.5 (Fig. 6d).

**Fig. 7:**
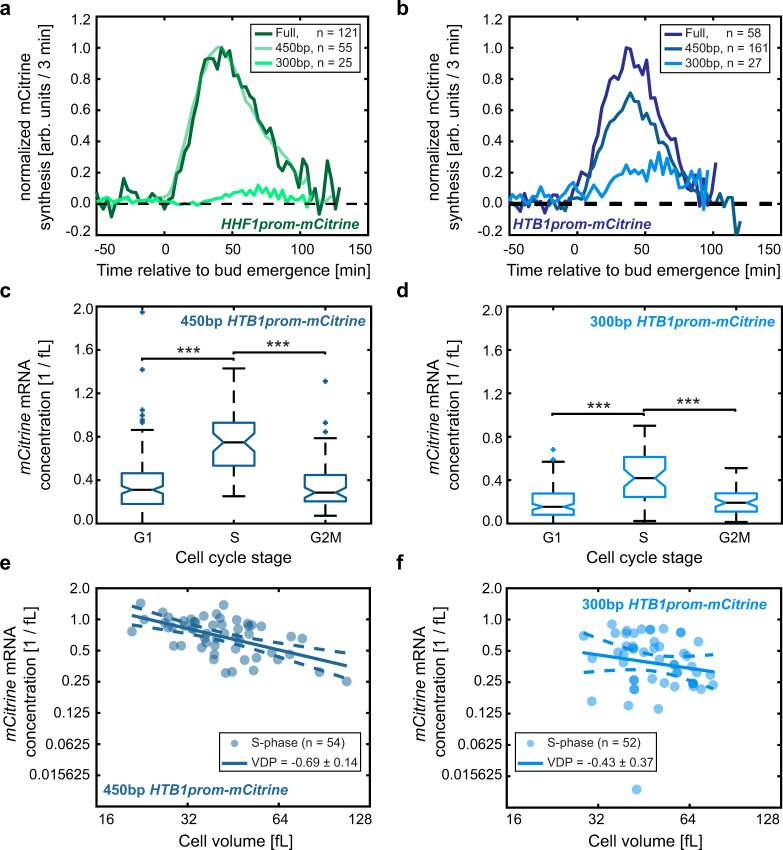
Change in behavior of truncated histone promoters is not due to a disruption of the cell-cycle-dependence. **a**, **b** *mCitrine* synthesis rate measured by live-cell fluorescence microscopy during the first cell cycle of new-born diploid cells, when expressed from *HHF1* (**a**) or *HTB1* (**c**) promoter truncations. Traces represent the mean of the moving averages over three frames of the single cell traces and are shown for the time span during which at least ten single cell traces were included in the average. All traces are aligned at the time of first bud emergence (*t* = 0) and normalized to the maximum mean value of *mCitrine* synthesis for the full promoter. **c**, **d** *mCitrine* mRNA concentration in G1-, S-, or G2/M-phases, estimated as the number of mRNA spots detected with smFISH in the whole cell including the bud and divided by the cell volume, measured for diploid cells expressing *mCitrine* from an additional 450 bp (**c**) or 300 bp (**d**) *HTB1* promoter truncation. Colored boxes highlight the 25- and 75-percentiles, whiskers extend to $\pm 2.7\sigma$ of the distributions and colored crosses highlight outliers. Black, horizontal lines indicate the median between single cells for ${n}_{{\rm{G}}1}\,=\,160,{n}_{{\rm{S}}}\,=\,54,{n}_{{\rm{G}}2{\rm{M}}}\,=\,66$ (**c**) and ${n}_{{\rm{G}}1}\,=\,131,{n}_{{\rm{S}}}\,=\,52,{n}_{{\rm{G}}2{\rm{M}}}\,=\,55$ (**d**), with notches indicating the 95% confidence interval. Significances were tested using a Kruskal–Wallis test at a confidence level $\alpha \,=\,0.05$; ***${p}_{{\rm{G}}1{\rm{vs}}\;{\rm{ S}}}\,=\,2.5\cdot {10}^{-14}$, ***${p}_{{\rm{S}}\; {\rm{vs}}\;{\rm{ G}}2{\rm{M}}}\,=\,2.9\cdot {10}^{-12}$ (**c**), ***${p}_{{\rm{G}}1{\rm{vs}}\;{\rm{ S}}}\,=\,5.2\cdot {10}^{-13}$, ***${p}_{{\rm{S}}\;{\rm{ vs}}\; {\rm{G}}2{\rm{M}}}\,=\,1.6\cdot {10}^{-8}$ (**d**). **e**, **f** *mCitrine* mRNA concentration in S-phase cells, expressed from an additional 450 bp (**e**) or 300 bp (**f**) *HTB1* promoter truncation, shown as a function of cell volume in a double-logarithmic plot. Solid lines show linear fits to the double-logarithmic data, dashed lines represent the 95% confidence intervals of the fit. Volume-dependence parameters (VDPs) were determined as the slope of the fit, with respective standard error.

To test that this switch in behavior is not due to a disruption of the cell-cycle-dependence, we analyzed *mCitrine* expression from the histone-promoter truncations with live-cell microscopy. As expected, total *mCitrine* intensity strongly decreases in the 300 bp compared to the 450 bp truncations. However, while the peak of mCitrine synthesis seems to be delayed for the 300 bp truncations of the *HTB1* and *HHF1* promoters, they both still show a clear peak of mCitrine synthesis after bud emergence (Fig. 7a, b). In addition, we did not observe a dependence of the mCitrine production period on cell volume for any of the *HTB1* and *HHF1* promoter truncations investigated (Supplementary Fig. 9a–d). This suggests that even though the 300 bp truncations of the *HTB1* and *HHF1* promoters have an effect on the level and exact timing of *mCitrine* expression, its cell-cycle dependence remains largely intact.

To further test that the switch in promoter behavior is caused by a change in expression during S-phase rather than a change in the cell-cycle-dependence, we performed smFISH to quantify *mCitrine* transcripts expressed from the 450 and 300 bp *HTB1* promoter truncations. Consistent with the live-cell microscopy results, we find that both promoter truncations show a peak of expression during S-phase (Fig. 7c, d, Supplementary Fig. 9e, f). Moreover, we find that the transcript concentration in S-phase cells significantly decreases with cell volume for the 450 bp—but not for the 300 bp—promoter truncation (Fig. 7e, f). In summary, while we cannot fully exclude that differences in the cell-cycle dependences might contribute to the switch in behavior of the transcript concentrations, this would appear unlikely to fully account for the observed change.

### Transcriptional feedback might contribute to the cell-volume-dependent regulation by the *HTB1* promoter

In summary, our analysis of the histone-promoter truncations suggests that decreasing promoter strength can shift the volume- and ploidy-dependence of the histone promoters to an actin-like behavior, as predicted by a minimal model. Consistent with such a picture, both the *HTB1* and the *HHF1* promoters include well-characterized UASs partially located in the 150 bp sections that are lost between the 450 bp and 300 bp truncations (Supplementary Fig. 8a). These UAS elements act as binding sites for the transcription factor Spt10, which activates histone transcription during S-phase^36^. It is therefore plausible that the partial loss of the UAS elements causes the reduction in promoter strength observed for the 300 bp truncations, which in the model is described as a reduced initiation rate. However, in the case of the *HTB1* promoter, the section lost for the 300 bp truncation also includes the NEG element^38,39^, which is necessary for HIR-dependent negative feedback^40^ (Supplementary Fig. 8a). While our smFISH and live-cell microscopy results (Fig. 7e, f, Supplementary Fig. 9c, d) suggest that the cell-volume-dependence of *HTB1* promoter driving transcription is not due to a change in the S-phase duration, transcriptional feedback sensing the amount of histone protein could in principle still account for the cell-volume-dependence if it acts uniformly throughout S-phase while DNA is replicated. In this case, loss of the NEG element provides an alternative explanation for the change to actin-like behavior observed for the truncated *HTB1* promoter.

To examine a potential role of NEG-mediated feedback, we deleted *HIR1* in the strain carrying the additional *HTB1* promoter. Similar to the effect on the endogenous *HTB1* (Fig. 2f), we found that deleting *HIR1* results in a significantly weaker decrease of *mCitrine* transcript concentration with cell volume (Supplementary Fig. 10a, b). Surprisingly, even though the *HTB2* promoter does not include an NEG element and is therefore not thought to be subject to HIR-dependent regulation, we also observed a similar effect on the cell-volume-dependence of *mCitrine* expression when we repeated the experiment in the strain carrying the additional *HTB2* promoter (Supplementary Fig. 10c, d). Thus, while transcriptional feedback regulation might contribute to the cell-volume-dependence mediated by the *HTB1* promoter, we cannot exclude that the observed weaker cell-volume-dependence is due to an indirect effect of deleting *HIR1*.

**Fig. 8:**
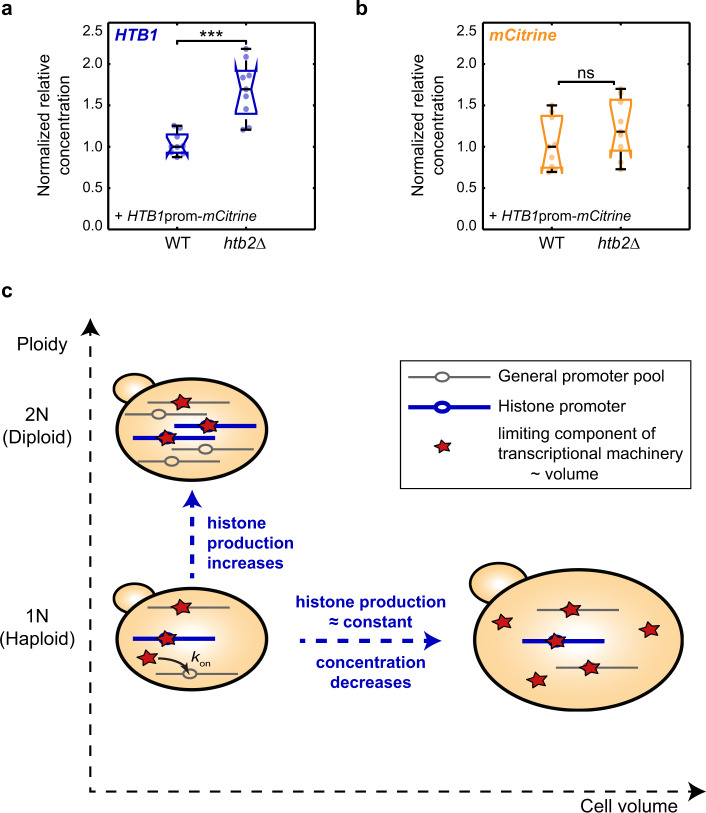
Histone promoters can couple gene expression to genome content. **a**, **b** Relative *HTB1* (**a**) and *mCitrine* (**b**) mRNA concentrations (normalized on *RDN18*) for a wild-type haploid strain carrying an additional *HTB1* promoter driving *mCitrine* expression, and a *htb2∆* in the same background, measured by RT-qPCR. Concentrations are normalized on the respective median concentration in the wild-type. Biological replicates are represented as colored data points (circles, $n$ = 9), colored boxes highlight the 25- and 75-percentiles and whiskers extend to the minimum and the maximum of the distributions. Black, horizontal lines indicate the median of the biological replicates, notches indicate the 95% confidence interval. Significances were tested using a two-tailed, two-sample *t* test at a confidence level $\alpha \,=\,0.05$; ***${p}_{{\rm{HTB}}1}\,=\,3.5\cdot {10}^{-4}$. **c** Illustration of the mechanism identified in this study. Through template-limited transcription, cells can quantitatively couple histone production to DNA content even though total biosynthetic capacity is linked to cell volume. This results in a decrease of histone concentration with increasing cell volume and an increase with increasing ploidy.

By deleting *HTB2* in the haploid strain that carries the additional *HTB1* promoter driving expression of *mCitrine*, we then further tested whether the *HTB1* promoter exhibits transcription-based dosage compensation. As before (Supplementary Fig. 5b), we observe a significant overexpression of endogenous *HTB1* upon deletion of *HTB2* (Fig. 8a). However, we do not observe a significant increase of *mCitrine* transcript concentration (Fig. 8b), which was surprising given that an *HTB1* promoter reporter construct containing the Htb1 N-terminus was reported to show dosage compensation upon deletion of the full *HTA2-HTB2* locus^21^. Taken together, our results indicate a contribution of *HIR1*-dependent regulation on the cell-volume-dependence of histone expression, but suggest that even in the case of the *HTB1* promoter, the observed decrease of transcript concentration with cell volume is not fully due to feedback regulation on the transcript level.

## Discussion

Taken together, we identified a mechanism that allows cells to deal with a fundamental challenge—how to quantitatively couple histone production to DNA content even though total biosynthetic capacity is linked to cell volume instead (Fig. 8c). We found that this coordination is already achieved at the transcript level. This finding was recently also confirmed by independent RNA-seq analysis of differently sized cell populations obtained by a combination of centrifugal elutriation with a synchronous release from a G1 arrest^41^. While mRNA degradation and feedback mechanisms contribute to histone homeostasis, our results suggest that competition for potentially limiting transcriptional machinery can be sufficient to achieve differential regulation of histone and other transcript concentrations with cell volume and ploidy. Specifically, if transcription is limited by the availability of limiting machinery, larger cells with more machinery will produce proportionally more mRNA, maintaining constant transcript concentrations. Since each gene will compete for the limiting machinery, transcription rate per gene decreases in inverse proportion with ploidy. Since the number of gene copies increases with ploidy, the total transcription rate is then independent of ploidy at a given cell volume. If transcription is instead limited by the gene itself, transcript concentrations will decrease with cell volume but will be proportional to ploidy because each individual gene copy will exhibit a transcription rate independent of ploidy context.

It was recently proposed that mRNA degradation in budding yeast is modulated dependent on cell volume^25^. While mRNA degradation is well known to contribute to histone homeostasis, it seems unlikely that degradation is responsible for the cell-volume dependence we observe for histone promoters expressing *mCitrine*. This is because we observe different cell-volume dependences for the 300 bp promoter truncations compared to the full promoters of *HTB1* and *HHF1*. This excludes the possibility that degradation controlled by the 5′-untranslated regions of the histone genes, which is included in all promoter truncation constructs we studied, is responsible for the histone-specific coupling of transcriptional output to DNA content rather than cell volume. However, we cannot fully exclude the possibility that the untranscribed part of the promoter indirectly controls mRNA degradation through an “imprinting” mechanism^42^.

Our work identifies a general mechanism that can be sufficient to couple histone amounts to DNA content. However, it also suggests that the exact regulation varies between the individual histone genes. Specifically, our results indicate that for the *HTB1* promoter, feedback regulation at the transcriptional level might contribute to the coupling of histone homeostasis to DNA content. Future work will therefore be needed to disentangle the specific contributions to the regulation of each individual histone gene.

In addition to histones, other proteins will require differential regulation with cell volume. For example, the G1/S inhibitors Whi5 in yeast^18^ and Rb in mammalian cells^43^ have recently been shown to decrease in concentration with cell volume, enabling cells to sense and control their size. Along those lines, a recent study suggested that many cell-cycle regulators show differential transcriptional regulation with cell volume^44^. The simplicity of template-limited transcription therefore suggests that it may be broadly employed across species to differentially regulate the concentrations of larger subsets of proteins, in particular to couple the amount of DNA-binding proteins to DNA content. Moreover, in addition to the ideal template- or machinery-limited regimes, cells can achieve a large variety of cell volume- and ploidy dependences, which importantly can be decoupled from the expression level of a given gene by independently tuning its initiation and elongation rates. Specific regulation of mRNA and protein degradation provides yet another level of control that cells can employ to tune the dependence of protein concentrations on cell volume and ploidy. In fact, our observation that the cell-volume-dependence of histone transcripts is even stronger in *rrp6* deletion cells cannot be explained by our simple model and suggests that such additional regulation contributes to cell-volume-dependent histone homeostasis in budding yeast. To quantitatively understand the cell volume- and ploidy-dependence of protein homeostasis on a genome wide level, it will therefore be crucial to identify the rate-limiting steps of transcription and mRNA degradation as well as the corresponding rate-limiting molecules.

## Methods

### Yeast strains

All yeast strains used in this work are based on W303 and were constructed using standard methods. Full genotypes of all strains are listed in Supplementary Table 1.

### Inducible-Whi5 strain

In order to increase the range of observable cell volumes, we used strains with β-estradiol-inducible *WHI5*, similarly described in previous works^18,45^. For this purpose, we deleted the endogenous alleles of the G1/S inhibitor *WHI5* and integrated one copy of *WHI5* expressed from an artificial, β-estradiol-inducible promoter system^19^. Specifically, this inducible promoter system consists of a β-estradiol-dependent, artificial transcription factor, which can bind an artificial promoter. This promoter is then used to induce *WHI5* expression.

To ensure that β-estradiol addition itself has no effect on cell growth, we grew cell cultures of a non-inducible *WHI5* haploid strain and cell cultures of a *whi5Δ* haploid strain, containing the β-estradiol-dependent, artificial transcription factor, but no copy of *WHI5*. We then added β-estradiol to those cultures and quantified the mean cell volumes after 24 h of growth in the presence of β-estradiol, by measuring the cell volume distributions using a Coulter counter (Beckman Coulter, Z2 Particle Counter). Finally, we compared the mean cell volumes to the mean cell volumes obtained from cell populations without β-estradiol addition (Supplementary Fig. 3a). In addition, we performed reverse-transcription-qPCR (RT-qPCR) on cell populations with and without β-estradiol addition and compared the obtained mean values for several genes (Supplementary Fig. 3b, c). For the non-inducible *WHI5* haploid strain, we could not identify a significant deviation of the population means between the cell populations with and without β-estradiol addition. For the *whi5Δ* haploid strain, containing only the β-estradiol-dependent, artificial transcription factor, we observed a slight but significant reduction of the relative mean mRNA concentrations of *HTA2, HHF2* and *HHO1* at 30 nM compared to 0 nM β-estradiol, which was consistent with a slightly increased mean cell volumes at 30 nM β-estradiol. In contrast, performing the same experimental procedure on cell cultures of an inducible *WHI5* haploid strain leads to much stronger changes of mean cell volumes and relative mean mRNA concentrations for all histone genes, demonstrating that the observed decrease of histone mRNA concentrations is specific to the Whi5-dependent cell volume increase (Supplementary Fig. 3a, d).

### Live-cell fluorescence microscopy

Cultures (3 mL) were grown at 30 °C in synthetic complete media containing 2% glycerol and 1% ethanol (SCGE) for at least 6 h in a shaking incubator at 250 rpm (Infors, Ecotron). Appropriate β-estradiol concentrations were then added to inducible cells (0 nM and 30 nM for haploids or 50 nM for diploids) and the cultures were grown for at least 24 h to ensure steady-state conditions. Optical densities were measured using a spectrophotometer (Perkin Elmer, Lambda Bio+) and ${{\mathrm{OD}}}_{600} \,<\, 1.0$ was maintained through appropriate dilutions during culture growth. For imaging, 1 mL of cells (${{\mathrm{OD}}}_{600} \,<\, 1.0$) was spun down at 10k g-force for 1 min (Thermo Fisher Scientific, Pico 17), resuspended in 200 µL SCGE and sonicated for 5 s (Bandelin electronics, HD2070 and UW2070). 100 µL of this cell suspension was then introduced in a Cellasic microfluidics Y04C (haploids and non-induced diploids) or Y04D (induced diploids) plate. Note that no β-estradiol was used in the microfluidic device during the microscopy experiments, resulting in a gradual decrease of cell volume of induced cells after the start of the experiment.

Live-cell fluorescence microscopy experiments were performed on a Zeiss LSM 800 microscope (software installed: Zen 2.3, blue edition) with additional epifluorescence setup using a Cellasic microfluidics device to ensure constant media (SCGE) flow in the microfluidics plate throughout the experiment. Experiments ran for 12 h with images being taken every 3 min using an automated stage (WSB Piezo Drive Can), a plan-apochromat 40×/1.3 oil immersion objective and an axiocam 506 camera. Phase-contrast images were taken at an illumination voltage of 4.5 V and an exposure time of 30 ms. *mCitrine* images were taken using the Colibri 511 LED module at 25% power and an exposure time of 10 ms. For each condition, at least two independent biological replicates were measured on different days. For experiments performed on cells with fluorescently tagged *HTB1* and cells carrying an additional promoter driving *mCitrine* expression, a microscope maintenance service had to be performed between imaging of biological replicates, which resulted in increased illumination intensities. Imaging parameters for the *mCitrine* channel were adjusted to avoid photo toxicity: images were taken using the Colibri 511 LED module at 5% power and an exposure time of 100 ms.

To correct for inaccuracies of the x–y-stage between time points, movies were first aligned using a custom Fiji^46^ script. Then, cell segmentation and quantification of the fluorescent signal as well as subtraction of background fluorescence and cell-volume-dependent autofluorescence (determined from control strains not expressing a fluorescent protein), and determination of time points of cell birth, bud emergence, and cytokinesis were performed with MATLAB 2017b using previously described methods^17,18,47^. For our analyses, we only included cells born during the experiment. Total fluorescence intensity after background- and autofluorescence correction was used as a proxy for total protein amount.

In order to determine total protein concentrations as total protein amounts divided by cell volume, we calculated cell volumes based on phase-contrast images. Briefly, after segmentation, cell areas were aligned along their major axis. We then divided the cells into slices perpendicular to their major axis, each 1 pixel in width. To estimate cell volume, we then assumed rotational symmetry of each slice around its middle axis parallel to the cell’s major axis, and summed the volumes of each slice to obtain total cell volume. This allowed us to analyze protein amounts and protein concentrations as a function of cell volume.

### Estimation of cell-cycle phases and histone production period using live-cell microscopy

To test whether the decrease of histone concentrations with cell volume could be explained by a decrease in the S-phase duration, and thus a shorter time period during which histones are produced, we aimed to estimate the duration of the histone production period (H-period; referred to as mCitrine production period for strains in which a histone promoter is driving *mCitrine* expression) from the mCitrine fluorescent intensity traces. For each single cell, we first performed a constant linear fit in each of the two plateaus of the fluorescence intensity, linked to G1- or G2/M-phase, respectively, and denoted them as ${P}_{1}$ and ${P}_{2}$. ${P}_{1}\,$ was obtained by performing the linear fit through the data points of the fluorescent intensity trace from cell birth to first bud emergence, ${P}_{2}$ was obtained by performing the linear fit through the last 30 min of the fluorescent intensity trace. We then set a threshold of 5%, determined the last time point for which $\,{I}_{{\mathrm{mCitrine}}} \,<\, {P}_{1}\,+\,0.05\,\cdot\, {P}_{1}$, and defined this time point as the beginning of the H-period. Similarly, we defined the first time point for which ${I}_{{\mathrm{mCitrine}}} \,> \, {P}_{2}\,-\,0.05\,\cdot\, {P}_{2}$ as the end of the H-period. Finally, the duration of the H-period was calculated as the difference between those two time points. We defined G1-phase duration as the time from cell birth to first bud emergence, and G2/M duration as the time between the end of the H-period and cytokinesis. Cells for which this approach failed where excluded from the cell-cycle phases analysis.

### Normalization of single cell fluorescent intensity traces after microscope maintenance service

In order to pool experimental data from two biological replicates imaged before and after maintenance service, respectively (Figs. 1c, 4a–c,  7a, b, Supplementary Fig. 7a, b, Supplementary Fig. 9a–d), intensities of single cell traces for the experiments taken before maintenance service were normalized to the intensities of experiments performed after maintenance service. For this purpose, the mean ${P}_{1}$ of all single cell traces before and after maintenance service was calculated and a normalization factor $a,$ determined as:

1 $$a\,=\,{P}_{1,{\mathrm{mean}}}^{{\mathrm{After}}}/{P}_{1,{\mathrm{mean}}}^{{\mathrm{Before}}}$$

Single cell traces before maintenance service were then multiplied with $a$ and those normalized single cell traces then pooled with single cell traces obtained after maintenance service.

### Estimation of *mCitrine* synthesis peak using live-cell microscopy

To characterize the cell-cycle-dependence of transcription from full and truncated histone promoters, we estimated the *mCitrine* synthesis rates from the mCitrine fluorescence intensity traces. For this purpose, we calculated the difference in mCitrine intensity between frame ${x}_{{\mathrm{n}}\,+\,1}$ and frame$\,{x}_{{\mathrm{n}}}$, for each frame of the single cell traces, which corresponds to the mCitrine synthesis as a function of time. To remove measurement noise, we then calculated moving averages over three frames for the *mCitrine* synthesis curves. Finally, we calculated the mean of those smoothed single cell curves and show the mean for the time span during which at least ten single cell traces were included in the average.

### RNA extraction and RT-qPCR

Cultures (25 mL) were grown at 30 °C in yeast peptone media containing 2% glucose (YPD) for at least 6 h in a shaking incubator at 250 rpm, before being washed and transferred to SCGE. The cultures were grown for at least 16 h before appropriate β-estradiol concentrations were added to inducible cells (0, 10, and 30 nM). The cultures (final volume of 50 mL) were then grown for at least 24 h in order to ensure steady-state conditions. During culture growth, ${{\mathrm{OD}}}_{600} \,<\, 1.0$ was maintained through appropriate dilutions. Cell-volume distributions of the cultures were measured with a Coulter counter after sonication for 5 s.

Remaining cell cultures were spun down at 4000 rpm for 5 min and the cell pellet resuspended in 50 µL nuclease-free water (Qiagen). Total RNA was extracted using a hot acidic phenol (Sigma-Aldrich) and chloroform (Thermo Fisher Scientific) extraction method adapted from an established protocol^48^. Yield of RNA was increased by precipitation in 100% ethanol (Merck Millipore) at −20 °C overnight, followed by a second precipitation in 100% ethanol at −80 °C for 2–4 h. As a quality check for total RNA extraction, agarose gel electrophoresis (1% agarose gel, run 30 min at 100 V) was performed to check for the presence of the 25, 18, and 5.8 s ribosomal RNA bands. Concentration and purity of the RNA samples were measured with a spectrophotometer (Thermo Fisher Scientific, NanoDrop 2000) at 260 nm and 280 nm. cDNA was then obtained from 800 ng total RNA in a PCR cycler (Applied Biosystems, ProFlex PCR system 3 × 32-well) using random primers and a high-capacity cDNA reverse-transcription kit following the included protocol (Thermo Fisher Scientific).

Quantitative PCR (qPCR) measurements were carried out on a LightCycler 480 Multiwell Plate 96 (Roche) using a DNA-binding fluorescent dye (BioRad, SsoAdvanced Universal SYBR Green Supermix) and mRNA sequence specific primers (Sigma-Aldrich). The qPCR was performed with 2 µL of a 1:10 dilution of the cDNA for the genes *ACT1*, *HHO1*, *HTB2* and *mCitrine*, or a 1:100 dilution for all other genes. Melting curve data were analyzed to verify primer specificity. Each sample was measured in technical duplicates and the mean value ${C}_{{\mathrm{P}}}^{{\mathrm{Gene}}}$ was used for further analyses if ${\sigma }_{{C}_{{\mathrm{P}}}^{{\mathrm{Gene}}}} \,<\, 0.5$. Relative concentrations, normalized on the reference gene *RDN18* were calculated using the equation:

2 $${{{\log }}}_{2}\left({\mathrm{relative}}\; {\mathrm{concentration}}\right)\,=\,-\left({C}_{{\mathrm{P}}}^{{\mathrm{Gene}}}\,-\,{C}_{{\mathrm{P}}}^{{\mathrm{RDN}}18}\right)$$

In order to analyze relative concentrations as a function of cell volume, the mean cell volumes were determined from the measured cell volume distributions. For each condition measured, the RT-qPCR experiments were performed at least three times on different days.

### Test for qPCR primer specificity

To test the specificity of the qPCR primer used to quantify histone mRNA concentrations, we analyzed deletion strains, where possible, for their respective deleted gene to check for unspecific primer binding. For example, we performed a qPCR measurement with the *HHO1* primers on a *hho1∆* strain and compared the obtained ${C}_{{\mathrm{P}}}$values with the ${C}_{{\mathrm{P}}}$ values obtained in the reference strain MS63-1 (Supplementary Table 1). We constructed deletion strains for the genes *HHO1*, *HTB2*, *HHF1*, *HHF2*, *HHT1*, and *HHT2*, for which we obtained viable colonies without dramatic growth defects. RNA was extracted as described above, and 1 µg of total RNA was reverse-transcribed using the above mentioned high-capacity cDNA synthesis kit. The qPCR was performed with 2 µL of a 1:10 dilution of each cDNA sample, and measured in 3 or 6 technical replicates. ${C}_{{\mathrm{P}}}$ values and melting curve data were analyzed to verify primer specificity. Results are shown in Supplementary Table 2, deletion strains used are listed in Supplementary Table 1, a list of all qPCR primers used can be found in Supplementary Table 3.

### Flow cytometry

Cultures (2–5 mL) were grown in YPD for at least 6 h in a shaking incubator (30 °C, 250 rpm) before being washed and transferred to SCGE and grown for at least 16 h. Appropriate β-estradiol concentrations were then added to inducible cells (0 nM and 30 nM for haploids or 50 nM for diploids), and the cultures grown for at least 24 h in a final volume of 3–5 mL. During cell growth, ${{\mathrm{OD}}}_{600} \,<\, 1.3$ was maintained through appropriate dilutions.

Cell-volume distributions of cultures were measured with a Coulter counter after sonication for 5 s. Cells were fixed using a 37% formaldehyde solution (Sigma-Aldrich) by pipetting 100 µL of formaldehyde into 900 µL of cell cultures in order to achieve a final formaldehyde concentration of 3.7%. Cultures were incubated at room temperature on a rotator (VWR International, Tube Rotator) for 15 min, spun down at 10 k g-force for 3 min and subsequently washed and resuspended in 100–1000 µL 100 mM potassium phosphate (pH 7.5). Samples were then stored on ice until being used for flow cytometry.

Flow Cytometry measurements were carried out on a benchtop flow cytometer with octagon and trigon detector arrays (BD Biosciences, LSR II, software installed: BD FACSDiva 8.0.1). Strains expressing the fluorescent protein *mCitrine* were excited with a 488 nm coherent sapphire solid-state laser paired with a 530/30 nm filter set. Side-scatter voltage was set to 220 V for all measurements, voltages for forward-scatter and photomultiplier tubes were adjusted depending on whether haploid or diploid cells or both were being measured. However, identical settings were used for replicate experiments. After removing obvious outliers or potential doublets through standard gating strategies (Supplementary Fig. 11), at least 10.000 cells were measured in the final stopping gate. For each experiment, cells not expressing *mCitrine* were measured to determine the cell-volume-dependent autofluorescence background which was subtracted from the mean fluorescence intensity of each sample measured in the same experiment. In order to calculate fluorescence concentrations, mean cell volumes were determined from the cell volume distributions measured with the Coulter counter. Mean fluorescence concentrations were then calculated by dividing the mean fluorescence intensity of each sample by its mean cell volume, allowing us to analyze mCitrine fluorescence concentrations as a function of cell volume. For each condition measured, the flow cytometry experiments were performed at least three times on different days.

### Cell-cycle analysis using flow cytometry

To get insights into the distributions of cell-cycle phases in cell populations of non-inducible and inducible-*WHI5* haploid and diploid strains, we performed cell-cycle analysis using flow cytometry. For this purpose, cell cultures (5 mL) were grown in YPD for at least 6 h in a shaking incubator (30 °C, 250 rpm), before being washed and transferred to SCGE, where appropriate β-estradiol concentrations were added (10 nM or 30 nM for haploid cells, 50 nM for diploid cells). The cultures were then grown for at least 24 h, assuring ${{\mathrm{OD}}}_{600} \,<\, 1.3$ during culture growth through appropriate dilutions. Cell-volume distributions of cultures were measured with a Coulter counter after sonication for 5 s. To fixate the cells and subsequently stain the DNA, we followed an already established protocol^49^. Specifically, 1 mL of each cell culture was pipetted into 9 mL of cold 80% ethanol and incubated at 4 °C on a rotator overnight. The cultures were then spun down at 4000 rpm for 2 min and washed twice in 50 mM Tris-HCl (pH = 8.0). Cells were then successively treated with a 1 mg/mL RNase A (Thermo Fisher Scientific) solution for 40 min at 37 °C, a 20 mg/mL Proteinase K (Promega) solution for 1 h at 37 °C and a 10x SYBR Green I (Sigma-Aldrich) solution for 1 h at room temperature. Between each treatment, cells were washed twice with 50 mM Tris-HCl and resuspended in 50 mM Tris-HCl. After the last treatment, cells were sonicated for 5 s. Flow Cytometry measurements were carried out on the benchtop flow cytometer described above, using the same laser, filter sets and side-scatter voltage. Settings for forward-scatter and photomultiplier tubes were adjusted depending on the condition measured. To estimate cell-cycle fractions, imaged DNA content frequency histograms were analyzed using Watson modeling. However, we noticed that for cell populations with large cell volumes (i.e., high β-estradiol concentrations), the DNA content distributions showed pronounced tails at large cell volumes that were not fit by the model. We speculate that this tail represents an increased mitochondrial DNA content in large cells^50^, which suggests that a fraction of G1 cells would be wrongly identified as S phase. Thus, we decided to limit our analysis to classifying cells as either G1/S-phase or G2/M-phase (Supplementary Fig. 1c). Using this approach, we did not find a drastic influence of the β-estradiol concentration used for Whi5 induction on the cell-cycle distributions. For each condition measured, the experiments were performed two times on different days.

### Single-molecule fluorescence in situ hybridization (smFISH)

To detect individual mRNA molecules in single cells, we used commercially available Stellaris^®^ FISH probes. The custom probe set for the coding sequence of *mCitrine* was designed using the Stellaris^®^ FISH Probe Designer (Biosearch Technologies, available online at www.biosearchtech.com/stellarisdesigner) and consisted of 27 probes, each 18 nucleotides long and labeled with the fluorophore Quasar^®^—670 (Biosearch Technologies).

smFISH was carried out according to the Stellaris^®^ RNA FISH Protocol for *S. cerevisiae*, available online at www.biosearchtech.com/stellarisprotocols. Cultures (5 mL) were grown in YPD for at least 6 h in a shaking incubator (30 °C, 250 rpm) before being washed and transferred to SCGE. Those cultures were then grown overnight to reach ${{\mathrm{OD}}}_{600} \,\sim\, 0.25{{\,\mbox{-}}\,}0.4$ and fixed the next morning by adding 5 mL of 37% formaldehyde to 45 mL of cell culture (final concentration 3.7%) and incubating at room temperature for 45 min. After washing the cells twice with ice-cold fixation buffer (1.2 M sorbitol (Sigma-Aldrich), 0.1 M K2HPO4 (Sigma-Aldrich), pH 7.5), they were digested at 30 °C in 1 mL fixation buffer containing 6.25 µg zymolyase (Biomol). Progression of cell digestion was monitored using bright-field microscopy (VWR International, VisiScope BL114) and digestion was continued until most of the cells appeared dark, which was mostly the case after 55 min of incubation. The digested cells were then washed with ice-cold fixation buffer and stored at 4 °C in 70% EtOH overnight. For hybridization, 300 μL of digested cells were centrifuged, resuspended in 100 µL hybridization buffer (Stellaris^®^ RNA FISH Hybridization Buffer (Biosearch Technologies) with 10% v/v formamide (VWR International)) with a final Stellaris^®^ FISH probe concentration of 125 nM and hybridized overnight at 30 °C. Afterwards, cells were washed with wash buffer A (Stellaris^®^ RNA FISH 1X wash buffer A (Biosearch Technologies) with 10% v/v formamide), incubated in 1 mL of a DAPI solution (5 ng/mL DAPI in wash buffer A) at 30 °C for 30 min to stain the nuclear DNA and washed with Stellaris^®^ RNA FISH wash buffer B. For image acquisition, cell samples were mounted between glass microscopes slides (Thermo Fisher Scientific, Superfrost plus, 25 × 75 × 1 mm) and cover slides (VWR International, 18 × 18 mm No. 1) using Vectashield^®^ Mounting Medium (Vector Laboratories) and were allowed to settle overnight. Cells were imaged on a Zeiss LSM 800 microscope with additional epifluorescence setup using a 63×/1.4 oil immersion objective and an axiocam 506 camera. Stacks composed of 20 z-slices (0.24 µm step size) were acquired to cover the entire depth of cells. For each condition, multiple images were taken per experiment and at least two independent biological replicates were measured on different days. Before a microscope maintenance service, *mCitrine* images were taken using the Colibri 630 LED module at 55% power and an exposure time of 5 s. DAPI images were taken using the Colibri 385 LED module at 30% power and an exposure time of 130 ms. After microscope maintenance, *mCitrine* images were taken at 30% power and an exposure time of 5 s to roughly match the intensities in the images taken before. DAPI images were taken at 20% power and an exposure time of 80 ms. Bright-field images were consistently taken at an illumination voltage of 3 V and an exposure time of 100 ms.

### Quantification of mRNA spots with smFISH

To analyze the smFISH images and quantify single mRNA spots, we used FISH-quant v3^51^. Briefly, we first segmented the cells in the FISH-quant interface by manually tracing the outlines of the cells and the nuclei with the help of bright-field and DAPI images, respectively. In order to calculate cell volumes from the cell outlines, we then used the same method as described above by aligning the cells along their major axis, dividing them into slices perpendicular to their major axis, each 1 pixel in width, and then assuming rotational symmetry of each slice around its middle axis parallel to the cell’s major axis. Total cell volume was then obtained by summing the volumes of each slice.

To quantify single mRNA spots in the imaged cells, we used the batch processing tool of FISH-quant. First, we defined the ideal image filtering settings, resulting in images with little background and bright, localized spots, for each experiment by applying a Laplacian of Gaussian filter on one example image of each experimental condition. Quality of the filtered image was confirmed by visual inspection. Second, we performed a pre-detection of mRNA spots in this filtered example image to define the best intensity thresholds to use for the spot detection in the batch processing. We aimed to use example images containing at least one S-phase cell (with high number of mRNA spots). Finally, we analyzed all images belonging to the same experimental condition via FISH-quant batch processing. mRNA spots were detected and then fit with a 3D Gaussian on the raw, unfiltered images, allowing us to set different maximum thresholds for the spot sizes in xy and z, as well as a minimum threshold for the amplitude and intensity of the detected spots in order to differentiate background spots from real mRNA spots. Using this approach, most spots detected in a wild-type strain carrying no *mCitrine* allele were excluded, and we thus neglected the contribution of background in the mRNA *mCitrine* concentration for further analysis (Supplementary Fig. 7h). *mCitrine* mRNA concentration was estimated as the number of mRNA spots detected with FISH-quant in the whole cell including the bud, divided by the total cell volume including the bud.

### Classification of cell-cycle stages with smFISH

To classify cells in G1, S, or G2/M phase, we used the cell and nucleus segmentations performed with the help of bright-field and DAPI images. Cells having nuclear signal and no bud were classified as G1 cells, cells having a nuclear signal and a bud without nuclear signal were classified as S phase. However, if the ratio of bud area divided by mother area was greater than 0.3, the cells were classified as G2M cells instead. For cells having nuclear signal and a bud that also had nuclear signal, we ensured that they were still in G2M (rather than two separate G1 cells) by inspecting the bright-field and DAPI images.

### Volume-dependence parameter

Analyzing protein and mRNA concentrations as a function of cell volume reveals a decrease of concentration with increasing cell volume for histones. In order to quantify this decrease, we performed a linear regression on the double-logarithmic data and define the slope of the fit as the VDP:

3 $${{{\log }}}_{2}\left(c\right)\,=\,{{{{\log }}}_{2}}(c_{0})\,+\,{\mathrm{VDP}}\cdot {{{\log }}}_{2}\left(V\right)$$

The VDP gives us a quantitative measure for the relation of protein and mRNA concentrations with cell volume: a negative VDP indicates a decrease of concentration with increasing cell volume. The special case of VDP $\,=\,-1$ corresponds to a decrease of concentration with ${c} \,\sim\, 1/V$, and therefore signifies a constant amount of protein or mRNA with increasing cell volume. A positive VDP indicates an increase of concentration with increasing cell volume, and VDP $\,=\,0$ corresponds to a constant concentration ${{c}}_{0}$.

### Statistical analyses

#### Significance of VDPs

To test for a significant deviation of the VDP from 0, we performed two-tailed one-sample *t* tests on the regression coefficients of the linear fit at a confidence level of $\alpha \,=\,0.05.$ Our null hypothesis ${H}_{0}$ assumes the respective coefficient to be equal to 0. In order to test for the significance of the VDP, we are interested in the slope of the linear fit: for a *p* value smaller than $\alpha$, we reject ${H}_{0}$ and consider the slope, i.e., the VDP, to be significantly different from 0.

To test whether the VDPs of two different conditions significantly deviate from each other, we used a general linear regression model with a categorical variable, ${\mathrm{Type}}$, to differentiate between the two conditions analyzed:

4 $${{{\log }}}_{2}\left(c\right)\,=\,{{{{\log }}}_{2}}(c_{0})\,+\,{{\mathrm{VDP}}}_{0}\cdot {{{\log }}}_{2}\left(V\right)\,+\,{\delta }_{1}\cdot {\mathrm{Type}}\,+\,{\delta }_{2}\cdot {\mathrm{Type}}\cdot {{{\log }}}_{2}\left(V\right)$$

with$\,{c}_{0}$ and ${{\mathrm{VDP}}}_{0}$ corresponding to the reference condition $({\mathrm{Type}}\,=\,0)$, ${\delta }_{1}$ describing the average difference in the intercepts of the linear fits between the two conditions, and ${\delta }_{2}$ describing the change in the slopes (VDPs) between the two conditions. In order to test for a significant difference between the two VDPs, we perform a two-tailed one-sample *t* test on ${\delta }_{2},$ with the null hypothesis ${H}_{0}$ assuming ${\delta }_{2}$ = 0, at a confidence level of $\alpha \,=\,0.05$. For a *p* value smaller than $\alpha$, we reject ${H}_{0}\,$ and consider the change between the two slopes to be significant, i.e., we consider the two VDPs to be significantly different from each other.

#### Error estimation of concentrations at 60 fL

To calculate concentrations at a characteristic cell volume of 60 fL with respective error estimates, we evaluated the linear fits to the double-logarithmic data at 60 fL and estimated the 95% confidence intervals of the fit at 60 fL. When normalizing the concentration to a chosen value $x$, errors were calculated using error propagation:

5 $$\triangle y\,=\,y\cdot \sqrt{{\left(\frac{{\triangle c}^{2}}{{c}^{2}}\right)}^{2}\,+\,{\left(\frac{{\triangle x}^{2}}{{x}^{2}}\right)}^{2}}$$

with $y$ being the new normalized concentration and $c$ the previously calculated concentration.

To estimate the error associated with the ratio between the concentrations at 60 fL in haploids and diploids, we used bootstrap analysis. Specifically, we treated the measurements of protein or mRNA concentration and corresponding cell volume as a set of linked variables, both for haploid and diploid cells. We then resampled *n* = 10,000 populations of same size by random sampling with replacement from this experimental two-dimensional population. Next, we performed a linear regression on the double-logarithmic data for each of the resampled populations and estimated the concentration at 60 fL, giving us a distribution of *n* = 10,000 concentrations at 60 fL for both haploid and diploid cells. Finally, we randomly selected a concentration in each of those distributions, and divide the concentration for diploids by the concentration for haploids. We repeated this process 10,000 times with replacement to obtain a distribution of *n* = 10,000 concentration ratios, for which we calculate the median and the 2.5- and 97.5-percentiles.

#### Comparison of population means

When comparing distributions of mean cell volumes determined with a Coulter counter, or mRNA concentrations determined with either RT-qPCR or smFISH, we performed the following statistical tests to assess whether the population means were significantly different from each other. First, we performed a Shapiro–Wilk test at a confidence level of $\alpha \,=\,0.05$ to test whether the distributions were normally distributed. For normal distributions, we then performed a Bartlett test at a confidence level of $\alpha \,=\,0.05$ to test whether equal variances of the distributions could be assumed. If we could assume equal variances, we performed a two-tailed, two-sample *t* test assuming equal variances with the null hypothesis ${H}_{0}$ assuming equal means, at a confidence level of $\alpha =0.05$. If we could not assume equal variances, we performed a two-tailed, two-sample *t* test assuming unequal variances. For a *p* value smaller than $\alpha$, we reject ${H}_{0}$ and consider the means of the distributions to be significantly different from each other.

If we could not assume normal distributions, after performing the Shapiro–Wilk test, we performed a Kruskal–Wallis test with the null hypothesis ${H}_{0}$ assuming that our distributions are from the same population, at a confidence level of $\alpha \,=\,0.05$. For a *p* value smaller than $\alpha$, we reject ${H}_{0}$ and consider the distributions to not be from the same population.

### Minimal model

To obtain mechanistic insight on how the transcription rate of one specific promoter depends on cell volume and ploidy context, we sought to build a minimal model. Similar to Heldt et al.^37^ we consider transcription being limited by one component of the transcriptional machinery, potentially a subunit of the RNA polymerase. In addition, we assume transcript degradation to be the same for all transcripts, and set the corresponding degradation rate *k*_deg_ = 1, i.e., all other rates are normalized with respect to *k*_deg_. Note that in the case of stable transcripts, *k*_deg_ also describes dilution of transcripts by cell growth.

To account for the competition of different promoters for a finite number of the limiting component of the transcriptional machinery (${\mathrm{TM}}$), our model distinguishes two classes of promoters—a general pool of promoters, ${g}$, with ${n}_{{\mathrm{h}}}\,\approx\, 6000$ (haploids) or ${n}_{{\mathrm{d}}}\,\approx\, 12000$ (diploids), and a single promoter of interest, $p$, present as a single copy. We describe each promoter as one single binding site for ${\mathrm{TM}}$ and denote the number of TM bound to general promoters as ${{R}}^{g}$. Binding of ${\mathrm{TM}}$ at the single promoter of interest is described by ${{R}}^{{\mathrm{p}}}$, which can assume values between 0 (not bound) and 1 (bound). Moreover, ${R}^{{\mathrm{f}}}$ denotes the number of free ${\mathrm{TM}}$. We assume that the total number of ${\mathrm{TM}}$ (free and bound) scales proportionally to cell volume $V$ and is given by

6 $${R}^{g}\,+\,{R}^{{\mathrm{p}}}\,+\,{R}^{{\mathrm{f}}}\,=\,{c}_{\mathrm{TM}}V$$

with ${c}_{\mathrm{TM}}$ being the total ${\mathrm{TM}}$ concentration.

Assuming that the arrival of ${\mathrm{TM}}$ at promoters is proportional to the concentration of free $\,{\mathrm{TM}}$, the change in number of bound general promoters over time is given by following equation:

7 $$\frac{d{R}^{g}}{{dt}}\,=\,{k}_{\mathrm{on}}^{g}\left({n}_{{\mathrm{h}}/{\mathrm{d}}}\,-\,{R}^{g}\right)\frac{{R}^{{\mathrm{f}}}}{V}\,-\,{k}_{\mathrm{off}}^{g}{R}^{g}$$

where $\,{k}_{{on}}^{g}$ is the rate at which transcription is being initiated at each general promoter, ${n}_{{\mathrm{h}}/{\mathrm{d}}}\,-\,{R}^{g}$ are the number of general promoters not bound to ${\mathrm{TM}}$ in haploids or diploids, respectively, and ${k}_{\mathrm{off}}^{g}$ models the rate at which bound ${\mathrm{TM}}$ complete transcriptional elongation.

Similarly, the change in binding of TM to the single promoter of interest over time is given by:

8 $$\frac{d{R}^{{\mathrm{p}}}}{{dt}}\,=\,{k}_{{\mathrm{on}}}^{{\mathrm{p}}}\left(1\,-\,{R}^{{\mathrm{p}}}\right)\frac{{R}^{{\mathrm{f}}}}{V}\,-\,{k}_{\mathrm{off}}^{\mathrm{p}}{R}^{{\mathrm{p}}}$$

with parameters ${k}_{{\mathrm{on}}}^{{\mathrm{p}}}$ and ${k}_{\mathrm{off}}^{\mathrm{p}}$ representing transcriptional initiation and elongation, respectively, at the promoter of interest.

Solving (7) and (8) at steady-state $(\frac{d{R}^{g}}{{dt}}\,=\,\frac{d{R}^{{\mathrm{p}}}}{{dt}}\,=\,0)$, constraints the number of bound ${\mathrm{TM}}$s via the following nonlinear equations

9 $${k}_{{\mathrm{on}}}^{{\mathrm{g}}}\left({n}_{{\mathrm{h}}/{\mathrm{d}}}\,-\,{R}^{g}\right)\frac{{R}^{{\mathrm{f}}}}{V}\,=\,{k}_{\mathrm{off}}^{g}{R}^{g}$$

10 $${k}_{{\mathrm{on}}}^{{\mathrm{p}}}\left(1\,-\,{R}^{{\mathrm{p}}}\right)\frac{{R}^{{\mathrm{f}}}}{V}\,=\,{k}_{\mathrm{off}}^{\mathrm{p}}{R}^{{\mathrm{p}}}$$

Finally, the steady-state concentration of transcripts produced from the single promoter of interest is equal to ${{k}}_{{\mathrm{off}}}^{{\mathrm{p}}}{R}^{{\mathrm{p}}}/V$.

Given a set of parameters $\,{c}_{\mathrm{TM}},{k}_{\mathrm{on}}^{g},{k}_{\mathrm{off}}^{g},{k}_{{\mathrm{on}}}^{{\mathrm{p}}}\,{k}_{\mathrm{off}}^{\mathrm{p}}$, numerically solving Eqs. (6), (9) and (10) allows to calculate the transcript concentration, generated by the single promoter of interest as a function of cell volume $V$. We set ${c}_{\mathrm{TM}}\,=\,2000,{k}_{\mathrm{on}}^{g}\,=\,1,{k}_{\mathrm{off}}^{g}\,=\,{k}_{\mathrm{off}}^{\mathrm{p}}\,=\,3$ and calculate the steady-state concentration in haploids and diploids over cell volume for $\,{k}_{{\mathrm{on}}}^{{\mathrm{p}}}\,=\,\left[0.01,100\right]$.

In order to determine the VDP as a function of ${{k}}_{{\mathrm{on}}}^{{\mathrm{p}}}$, we calculated the concentration for each value of ${{k}}_{{\mathrm{on}}}^{{\mathrm{p}}}\,$ over a cell volume range of $V\,=\,\left[\frac{1}{3},3\right]$ and performed a linear regression fit on the logarithm of the concentration as a function of the logarithm of the cell volume, with cell volumes being equally spaced on the log scale. The VDP is then determined as the slope of the linear fit.

### Reporting summary

Further information on research design is available in the Nature Research Reporting Summary linked to this article.

## Supplementary information

Supplementary Information

Reporting Summary

## Data Availability

The data that supports this study is available from the corresponding author upon reasonable request. Source data are provided with this paper.

## Source data

Source Data
