# Supplementary material for: Transcription coordinates histone amounts and genome content
Source: Nat Commun. 2021 Jul 9;12:4202. doi: 10.1038/s41467-021-24451-8 (PMC8270936; doi:10.1038/s41467-021-24451-8)
Supplement: Supplementary file 1 — Supplementary Information [file 41467_2021_24451_MOESM1_ESM.pdf]

Extended Data figures

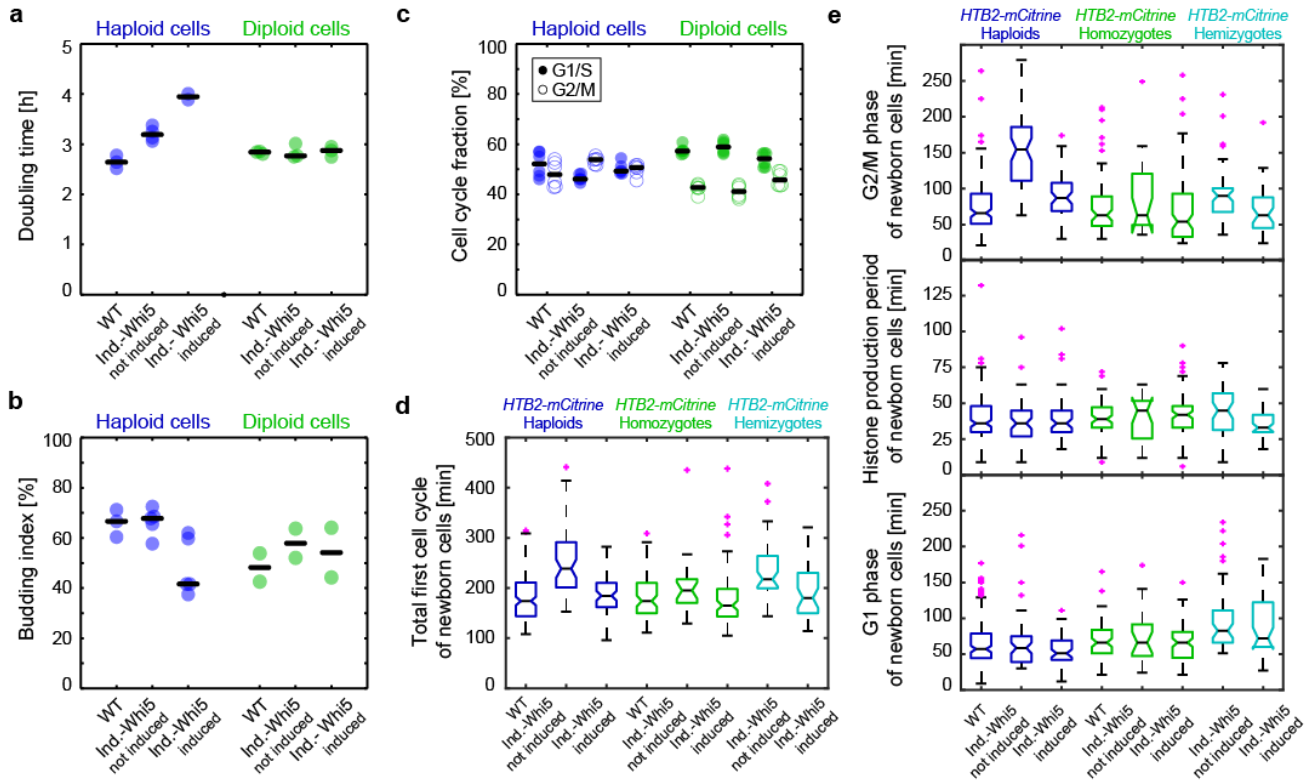

Supplementary Figure 1. Overexpression of Whi5 does not drastically alter doubling times, budding indices or cell cycle distributions. In all panels, haploid cells were induced with 30 nM β-estradiol, diploid cells with 50 nM. (a) Doubling times calculated from growth curves of exponentially growing cell populations for inducible and non-inducible (WT), untagged haploids (blue circles) and diploids (green circles). Black horizontal lines indicate the median of the distribution ($n_{\text{haploid}}^{\text{WT}} = 3$, $n_{\text{haploid}}^{\text{not ind.}} = 4$, $n_{\text{haploid}}^{\text{ind.}} = 2$, $n_{\text{diploid}}^{\text{WT}} = 3$, $n_{\text{diploid.}}^{\text{not ind.}} = 3$, and $n_{\text{diploid.}}^{\text{ind}} = 3$). (b) Budding index (percentage of budded cells), calculated by counting of budded and non-budded cells in exponentially growing cell populations using an optical microscope, for inducible and non-inducible (WT), untagged haploids (blue circles) and diploids (green circles). Black, horizontal lines indicate the median of the distribution ($n_{\text{haploid}}^{\text{WT}} = 3$, $n_{\text{haploid}}^{\text{not ind.}} = 5$, $n_{\text{haploid}}^{\text{ind.}} = 5$, $n_{\text{diploid}}^{\text{WT}} = 2$, $n_{\text{diploid.}}^{\text{not ind.}} = 2$, and $n_{\text{diploid.}}^{\text{ind}} = 2$). (c) Cell cycle distributions (percentage of cells in G1/S-phases (filled circles), or in G2/M-phases (open circles)) of inducible and non-inducible (WT), untagged haploid (blue) and diploid (green) cells, calculated from population distributions obtained through SYBR Green I staining of the DNA and flow cytometry. Black, horizontal lines indicate the median of the distribution ($n_{\text{haploid}}^{\text{WT}} = 6$, $n_{\text{haploid}}^{\text{not ind.}} = 6$, $n_{\text{haploid}}^{\text{ind.}} = 6$, $n_{\text{diploid}}^{\text{WT}} = 6$, $n_{\text{diploid.}}^{\text{not ind.}} = 6$, and $n_{\text{diploid.}}^{\text{ind}} = 6$). (d & e) Total first cell cycles of new-born cells (d) as well as individual G1- & G2/M-phases and

histone production periods of new-born cells (e) for inducible and non-inducible (WT) *HTB2-mCitrine* haploids (blue), *HTB2-mCitrine* homozygous diploids (green) and *HTB2-mCitrine/htb2Δ* hemizygous diploids calculated from single cell fluorescent traces, measured with live cell fluorescence microscopy. G1 phases are defined as the time from birth to the first budding, histone production phases as the time between the first point of increase in fluorescent signal and the last point of increase, G2/M phases as the time from end of histone production phase to the separation of the cell from its bud. Black, horizontal lines indicate the median between single cells ($n_{\text{haploid}}^{\text{WT}} = 145, n_{\text{haploid}}^{\text{not ind.}} = 58, n_{\text{haploid}}^{\text{ind.}} = 58, n_{\text{homoz}}^{\text{WT}} = 71, n_{\text{homoz.}}^{\text{not ind.}} = 21, n_{\text{homoz.}}^{\text{ind}} = 85, n_{\text{hemiz.}}^{\text{not ind.}} = 48$ and $n_{\text{hemiz.}}^{\text{ind.}} = 43$), with notches indicating the 95% confidence interval. Colored boxes highlight the 25- and 75-percentiles, whiskers extend to $\pm 2.7\sigma$ of the distribution and magenta crosses highlight outliers. Note that during image acquisition all cells were grown in the absence of β-estradiol.

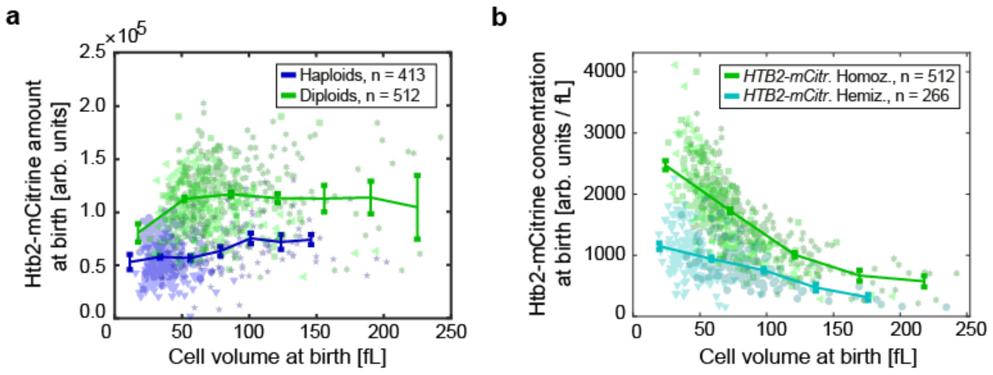

Supplementary Figure 2. Htb2-mCitrine amounts and concentrations increase with ploidy. (a) Htb2-mCitrine amounts at birth for *HTB2-mCitrine* haploids and *HTB2-mCitrine* homozygous diploids estimated from fluorescence microscopy as a function of cell volume. Individual data points for the different conditions are shown in blue for haploids (triangles for 0 nM, circles for non-inducible, stars for 30 nM) and green for diploids (triangles for 0 nM, squares for non-inducible, stars for 50 nM). Lines connect binned means with error bars indicating standard errors. (b) Htb2-mCitrine concentrations at birth for *HTB2-mCitrine* homozygous diploids and *HTB2-mCitrine/htb2Δ* hemizygous diploids as a function of cell volume. Individual data points for the different conditions are shown in teal for hemizygotes (triangles for 0 nM, circles for 50 nM) and green for homozygotes (triangles for 0 nM, squares for non-inducible, stars for 50 nM). Lines connect binned means with error bars indicating standard errors.

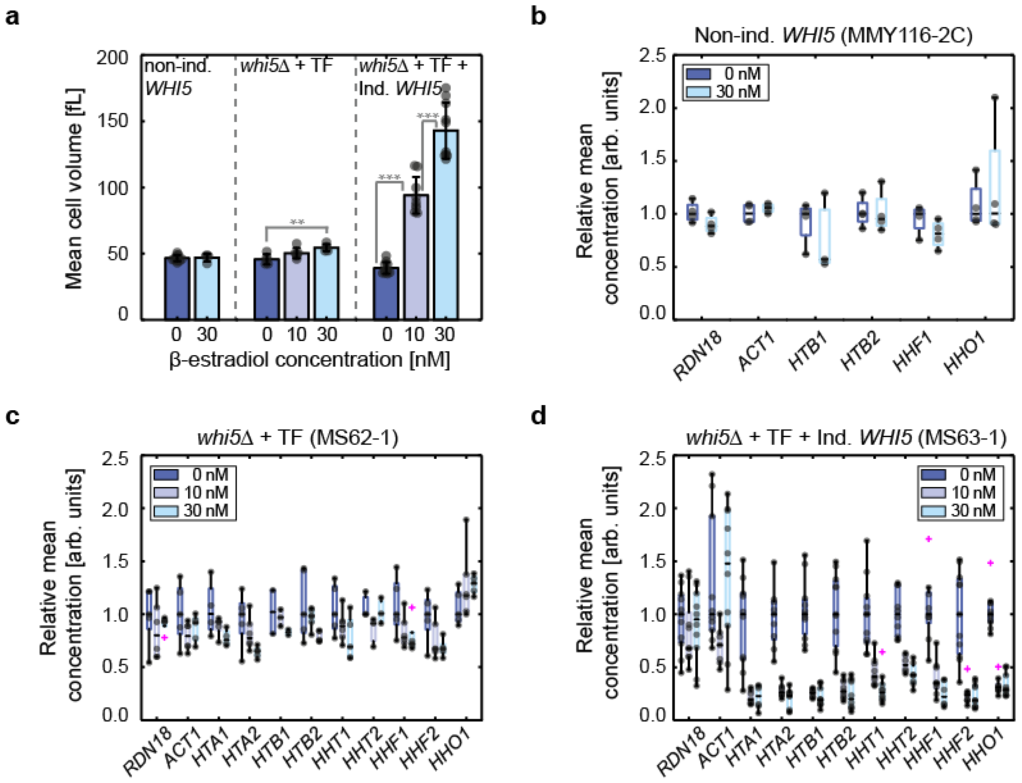

Supplementary Figure 3. Decrease in histone transcript concentration with cell volume is specific to Whi5-dependent cell volume increase. (a) Mean cell volumes (grey circles) of exponentially growing cell populations measured with a Coulter counter are shown as a function of β-estradiol concentrations for non-inducible haploid cells, *whi5Δ* haploid cells with β-estradiol-dependent transcription factor (TF), and *whi5Δ* haploid cells with β-estradiol-dependent transcription factor (TF) and β-estradiol-inducible *WHI5*. Bars show the mean of the population with error bars indicating the standard deviation for $n^{0}_{\mathrm{non-ind.}} = 11, n^{30}_{\mathrm{non-ind,}} = 4, n^{0}_{\mathrm{whi5\Delta+TF}} = 5, n^{10}_{\mathrm{whi5\Delta+TF}} = 6, n^{30}_{\mathrm{whi5\Delta+TF}} = 5, n^{0}_{\mathrm{Ind.-WHI5}} = 11, n^{10}_{\mathrm{Ind.-WHI5}} = 9, n^{30}_{\mathrm{Ind.-WHI5}} = 10$ biological replicates.

Significances were tested using a two-tailed, two-sample t-test at a confidence level $\alpha = 0.05$; $**p^{\mathrm{0nM\ vs\ 30nM}}_{\mathrm{whi5\Delta+TF}} = 3.2 \cdot 10^{-3}$, $***p^{\mathrm{0nM\ vs\ 10nM}}_{\mathrm{Ind.-WHI5}} = 8.3 \cdot 10^{-7}$, $***p^{\mathrm{10nM\ vs\ 30nM}}_{\mathrm{Ind.-WHI5}} = 2.0 \cdot 10^{-5}$. (b – d) Relative mean mRNA concentration (grey circles) of exponentially growing haploid cell populations with and without β-estradiol addition. (b) Non-inducible cells, (c) *whi5Δ* cells with β-estradiol-dependent transcription factor (TF), (d) *whi5Δ* cells with β-estradiol-dependent transcription factor (TF) and β-estradiol inducible *WHI5*. For each gene, values are normalized on the mean mRNA concentration of the cell populations without β-estradiol addition (0 nM). Black horizontal lines indicate the median of the distribution ($n = 4$ except for $n^{\mathrm{HTB1}}_{30} = 3$ (b), $n_0 = 5$, $n_{10} = 6$, and $n_{30} = 5$, except for $n^{\mathrm{HTA1}}_{0} = 4, n^{\mathrm{HTA1}}_{10} = 5, n^{\mathrm{HTA1}}_{30} = 4, n^{\mathrm{HTB1}}_{0} = 3, n^{\mathrm{HTB1}}_{10} = 3, n^{\mathrm{HTB1}}_{30} = 3, n^{\mathrm{HHT2}}_{0} = 3, n^{\mathrm{HHT2}}_{10} = 3$, and $n^{\mathrm{HHT2}}_{30} = 3$ (c), $n_0 = 11$, $n_{10} = 9$, and $n_{30} = 10$, except for

$n_0^{\mathrm{ACT1}} = n_0^{\mathrm{HTA1}} = n_0^{\mathrm{HHT2}} = 10, n_0^{\mathrm{HTA2}} = n_0^{\mathrm{HHF1}} = 9, n_{10}^{\mathrm{HTA2}} = n_{10}^{\mathrm{HTB1}} = 8, n_{10}^{\mathrm{HTA1}} = n_{10}^{\mathrm{HHT2}} = 6,$ $n_{10}^{\mathrm{ACT1}} = 7, n_{30}^{\mathrm{HTA2}} = 9, n_{30}^{\mathrm{HTA1}} = n_{30}^{\mathrm{HHT2}} = 7$ and $n_{30}^{\mathrm{HHF1}} = 6$ (d)). Grey boxes highlight the 25- and 75-percentiles, whiskers extend to $\pm 2.7\sigma$ of the distribution and magenta crosses highlight outliers.

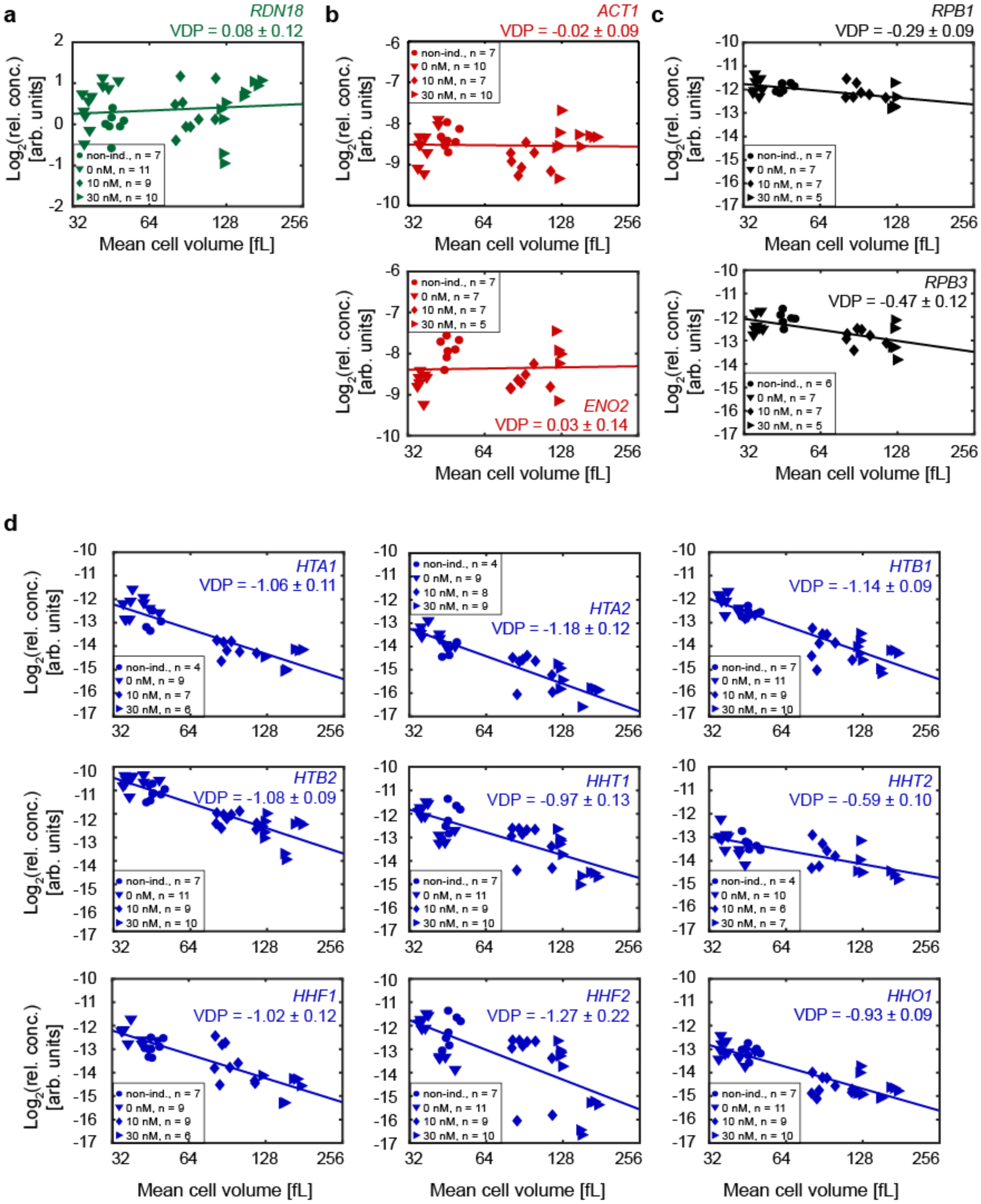

Supplementary Figure 4. Histone mRNA concentrations measured by RT-qPCR decrease with increasing cell volume. (a) Relative *RDN18* mRNA concentration for non-inducible and inducible haploid cells over mean cell volume, shown in a double logarithmic plot. mRNA concentrations are normalized on the mean mRNA concentration of non-inducible cells (green circles). (b – d) Raw data corresponding to Fig. 2d: Relative mRNA concentrations of *ACT1* and *ENO2* (b), *RPB1* and *RPB3* (b), as well as all core histone genes and *HHO1* (d) for non-inducible and inducible haploid cells over mean cell volume, shown in double logarithmic plots. mRNA concentrations are normalized on *RDN18* concentrations. For all panels, individual data points for the different conditions (down-

pointing triangles for 0 nM, circles for non-inducible, diamonds for 10 nM, left triangles for 30 nM) are shown in green (a), red (b), black (c) or blue (d). Lines show linear fits to the double logarithmic data, with volume-dependence parameters (VDPs) determined as the slope of the fit, with respective standard error.

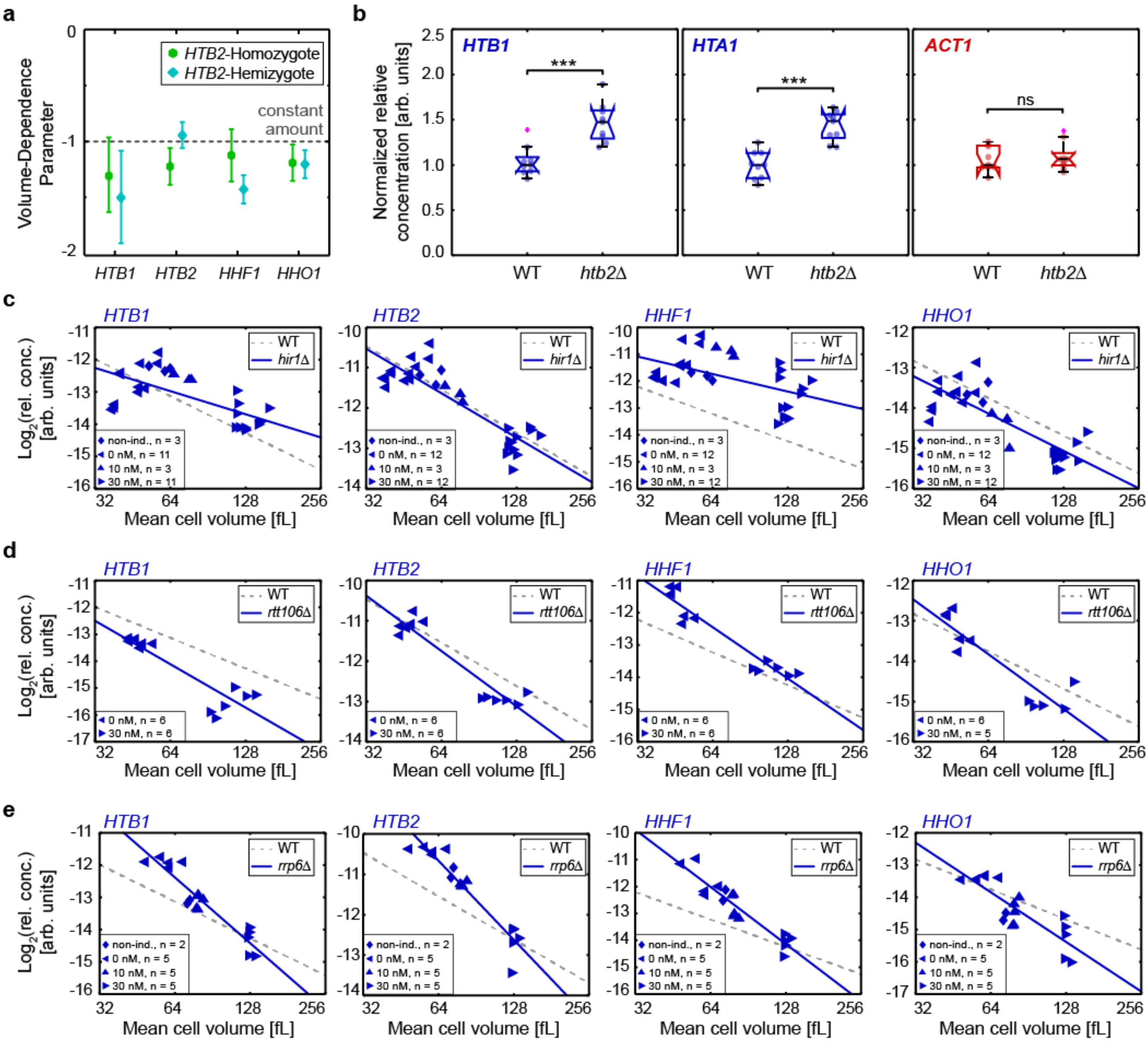

Supplementary Figure 5. Direct feedback, Hir1-dependent feedback, as well as 3'- to 5'-end degradation by the nuclear exosome is not necessary for the decrease of histone mRNA concentrations with cell volume. (a) Summaries of the VDPs for an *HTB2* homozygous (green circles) and *HTB2/htb2*Δ hemizygous diploid (teal diamonds), determined by RT-qPCR for *HTB1*, *HTB2*, *HHF1* and *HHO1*. VDPs were determined as the slopes of the linear fits to the double logarithmic concentration over cell volume data (fit through $n_{\mathrm{Homozygote}}^{\mathrm{HTB1,HTB2}} = n_{\mathrm{Hemizygote}}^{\mathrm{HTB1,HTB2}} = 18, n_{\mathrm{Homozygote}}^{\mathrm{HHF1,HHO1}} = n_{\mathrm{Hemizygote}}^{\mathrm{HHF1,HHO1}} = 12$ biological replicates). Error bars indicate the standard error of the slope. (b) Relative *HTB1*, *HTA1* and *ACT1* mRNA concentrations (normalized on RDN18) for a wild-type haploid strain and an *htb2*Δ in the same background, measured by RT-qPCR. Concentrations are normalized on the respective median concentration in the wild-type. Biological replicates are represented as colored data points (circles), colored boxes highlight the 25- and 75-

percentiles, whiskers extend to ±2.7σ of the distributions and magenta crosses highlight outliers. Black, horizontal lines indicate the median of the biological replicates (n = 9), notches indicate the 95% confidence interval. Significances were tested using a two-tailed, two-sample t-test at a confidence level $\alpha = 0.05$; $^{***}p_{\mathrm{HTB1}} = 2.6 \cdot 10^{-4}$, $^{***}p_{\mathrm{HTA1}} = 3.3 \cdot 10^{-5}$. (c – e) Raw data corresponding to Fig. 2f – h: Relative mRNA concentrations (normalized on *RDN18*) for inducible and non-inducible haploid cells over mean cell volume, shown in double logarithmic plots. Data corresponding to the *hir1*Δ (c), *rtt106*Δ (d) and *rrp6*Δ (e) cells are highlighted in blue for the different conditions (diamonds for non-inducible, left-pointing triangles for 0 nM, up-pointing triangles for 10 nM, and right-pointing triangles for 30 nM). Lines show the linear fits to the double logarithmic data. Grey dashed lines correspond to the linear fit for the wild-type cells, carrying no deletion, shown in Supplementary Fig. 4d.

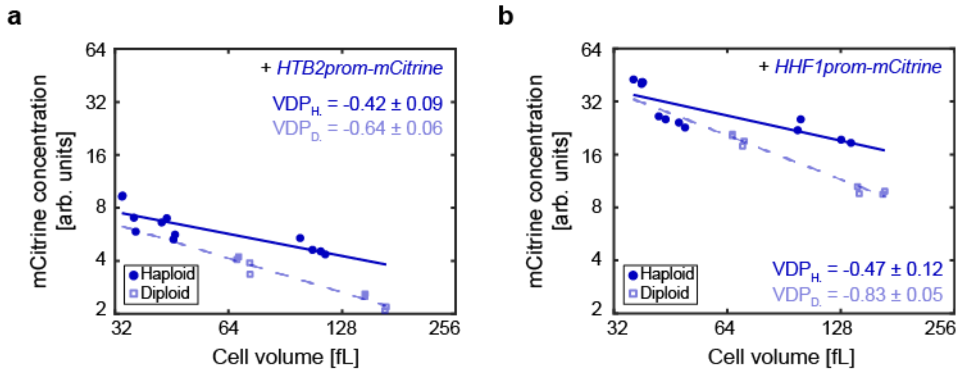

Supplementary Figure 6. Concentration of mCitrine expressed from a single additional histone promoter in haploid and diploid cells measured with flow cytometry. mCitrine concentration, driven by an additional copy of the *HTB2* (a) or *HHF1* (b) promoter in haploid (blue filled circles) and diploid (blue open squares) cells, shown as a function of cell volume in a double logarithmic plot. Lines show linear fits to the double logarithmic data with volume-dependence parameters (VDPs) determined as the slope of the fit, with respective standard error (fit through $n_{\text{Haploid}} = 12$ and $n_{\text{Diploid}} = 8$ biological replicates).

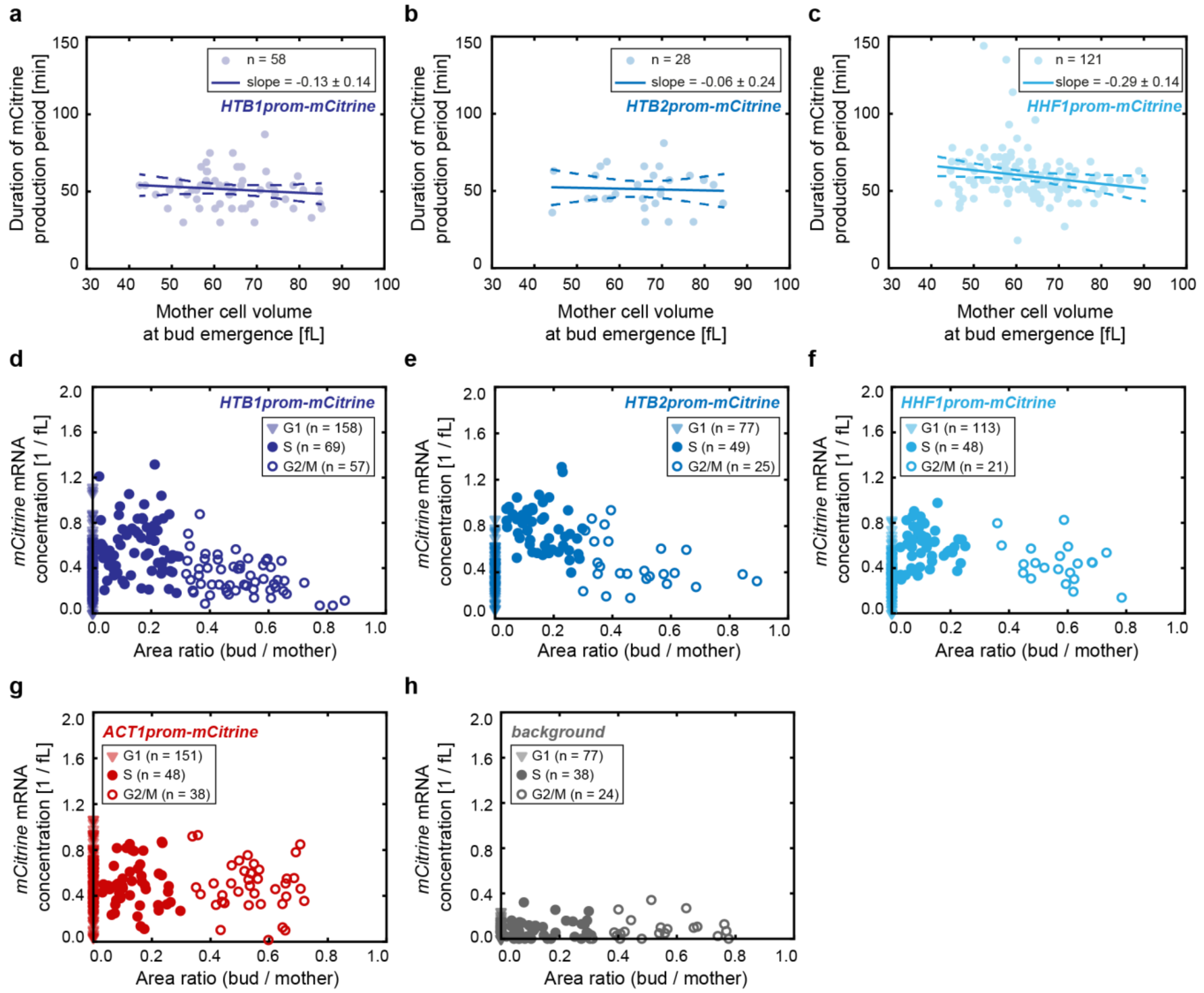

Supplementary Figure 7. Cell-cycle-dependence does not explain cell-volume-dependence of expression from histone promoters. (a – c) Duration of mCitrine production period, determined by live-cell fluorescence microscopy, shown as a function of the cell volume at bud emergence for a diploid strain carrying an additional *HTB1* (a), *HTB2* (b) or *HHF1* (c) promoter driving *mCitrine* expression. Solid lines show linear fits to the data, dashed lines represent the 95% confidence intervals of the fit. Slopes of the fit are stated with respective standard error. (d – h) *mCitrine* mRNA concentration, estimated as the number of mRNA spots detected with smFISH in the whole cell including the bud and divided by the cell volume, shown as a function of the cell area ratio (cell area of the bud divided by the cell area of the mother cell) for diploid cells expressing *mCitrine* from an additional *HTB1* (d), *HTB2* (e), *HHF1* (f) or *ACT1* (g) promoter and a wild-type diploid strain, carrying no *mCitrine* allele (h). Individual data points correspond to cells in G1-phase (down-pointing triangles), S-phase (filled circles) or G2/M-phase (open circles).

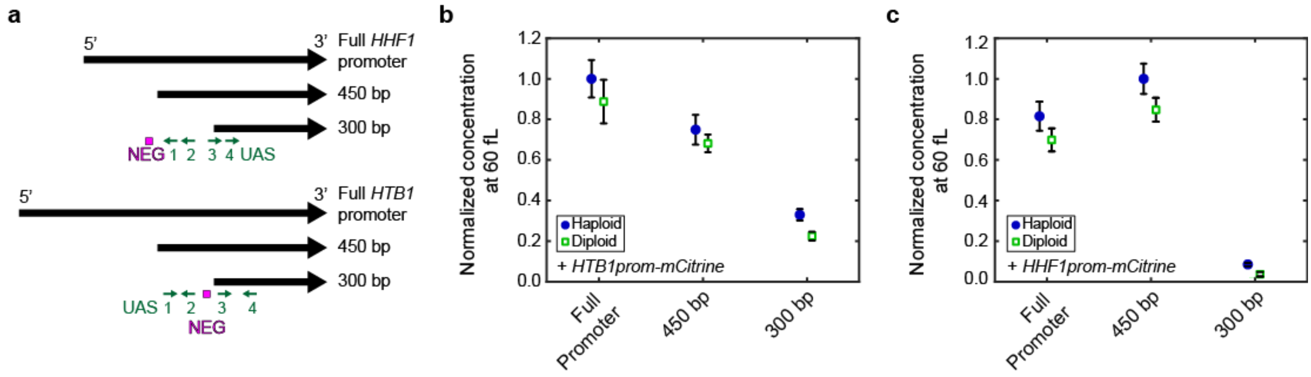

Supplementary Figure 8. mCitrine expression driven by single additional histone promoter truncations and measured by flow cytometry decreases once part of the upstream activating sequences (UASs) are truncated. (a) Illustration of the full *HHF1* and *HTB1* promoter, as well as the 450 bp and 300 bp truncations. Green arrows show the location of the upstream activating sequences (UASs)[35], magenta boxes show the location of the NEG elements[38,39]. (b - c) Normalized mCitrine concentration at 60 fL for different histone promoter truncations, integrated in haploid (blue filled circles) and diploid (green open squares) cells. (b) *mCitrine* driven by *HTB1* promoter truncations, (c) *mCitrine* driven by *HHF1* promoter truncations. Concentrations at 60 fL were calculated by the linear fit to the double logarithmic dependence of concentration on cell volume (fit through $n_{\mathrm{Haploid}}^{\mathrm{Full}} = n_{\mathrm{Haploid}}^{\mathrm{450bp}} = n_{\mathrm{Haploid}}^{\mathrm{300bp}} = 27,\ n_{\mathrm{Diploid}}^{\mathrm{Full}} = 18, n_{\mathrm{Diploid}}^{\mathrm{450bp}} = n_{\mathrm{Diploid}}^{\mathrm{300bp}} = 27$ and (b) $n_{\mathrm{Haploid}}^{\mathrm{Full}} = n_{\mathrm{Haploid}}^{\mathrm{450bp}} = n_{\mathrm{Haploid}}^{\mathrm{300bp}} = 27,\ n_{\mathrm{Diploid}}^{\mathrm{Full}} = n_{\mathrm{Diploid}}^{\mathrm{450bp}} = 18, n_{\mathrm{Diploid}}^{\mathrm{300bp}} = 17$ (c) biological replicates), and normalized to the maximum concentration calculated for haploid cells. Error bars are derived by error propagation of the 95% confidence interval of the linear fit at 60 fL.

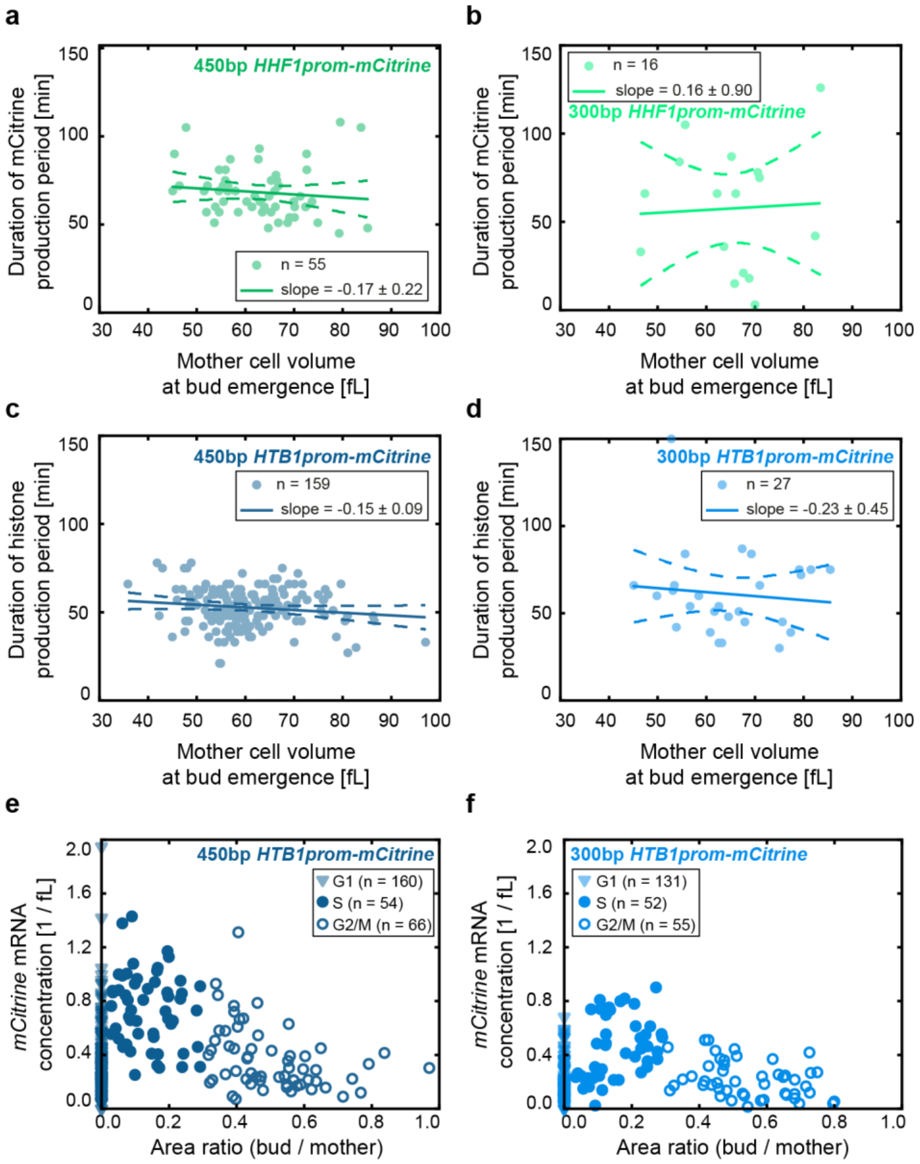

Supplementary Figure 9. Change in behavior of truncated histone promoters is not due to a disruption of the cell-cycle-dependence. (a – d) Duration of mCitrine production period, determined by live-cell fluorescence microscopy, shown as a function of the cell volume at bud emergence for a diploid strain carrying an additional 450 bp *HHF1* (a), 300 bp *HHF1* (b), 450 bp *HTB1* (c) or 300 bp *HTB1* (d) promoter truncation driving *mCitrine* expression. Solid lines show linear fits to the data, dashed lines represent the 95% confidence intervals of the fit. Slopes of the fit are stated with respective standard error. (e & f) *mCitrine* mRNA concentration, estimated as the number of mRNA spots detected with smFISH in the whole cell including the bud and divided by the cell volume, shown as a function of the cell area ratio (cell area of the bud divided by the cell area of the mother cell) for diploid cells expressing *mCitrine* from an additional 450 bp *HTB1* (e) or an additional 300 bp *HTB1* (f) promoter. Individual data points correspond to cells in G1-phase (down-pointing triangles), S-phase (filled circles) or G2/M-phase (open circles).

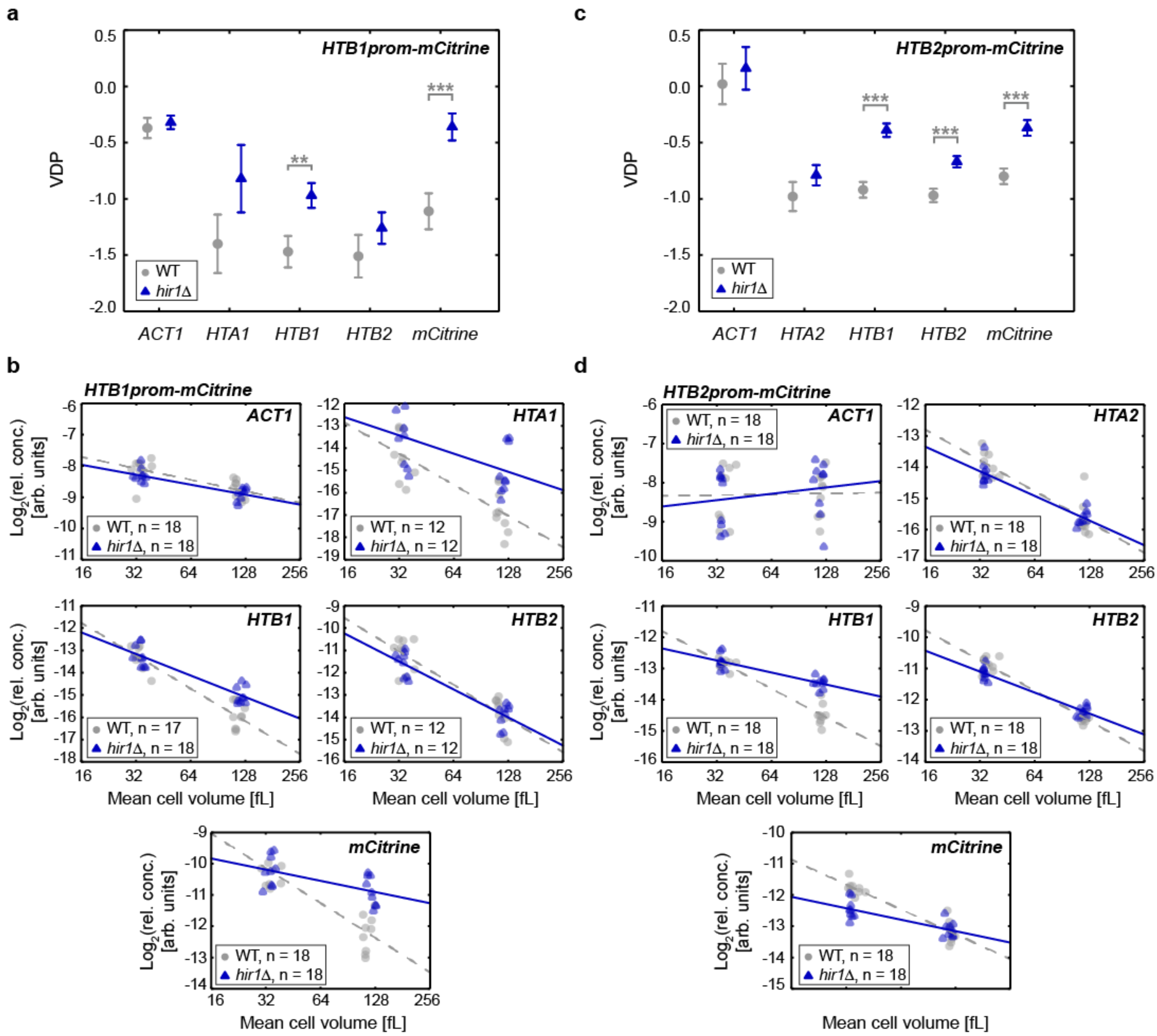

Supplementary Figure 10. *HIR1*-dependent regulation on the transcript level might contribute to the cell-volume-dependence of histone expression. (a – d) Summary of volume-dependence parameters (VDPs) (a & c) and corresponding raw data (b & d) for a haploid strain carrying an additional *HTB1* promoter (a & b) or an additional *HTB2* promoter (c & d) driving *mCitrine* expression (grey circles), and a *hir1*Δ (blue triangles) in the same background, determined by RT-qPCR for *ACT1*, *HTA1*/*HTA2*, *HTB1*, *HTB2* and *mCitrine*. VDPs (a & c) were determined as the slope of the linear fit (b & d) to the double logarithmic dependence of concentration on cell volume. Error bars indicate the standard error of the slope. Deviation of the VDP from that of the 'wild-type' (carrying no deletion) was tested using linear regressions; $^{**}p_{\mathrm{HTB1}} = 7.4 \cdot 10^{-3}$, $^{***}p_{\mathrm{mCitrine}} = 7.3 \cdot 10^{-4}$ (a), $^{***}p_{\mathrm{HTB1}} = 1.0 \cdot 10^{-6}$, $^{***}p_{\mathrm{HTB2}} = 9.2 \cdot 10^{-4}$, $p_{\mathrm{mCitrine}} = 2.4 \cdot 10^{-4}$ (c).

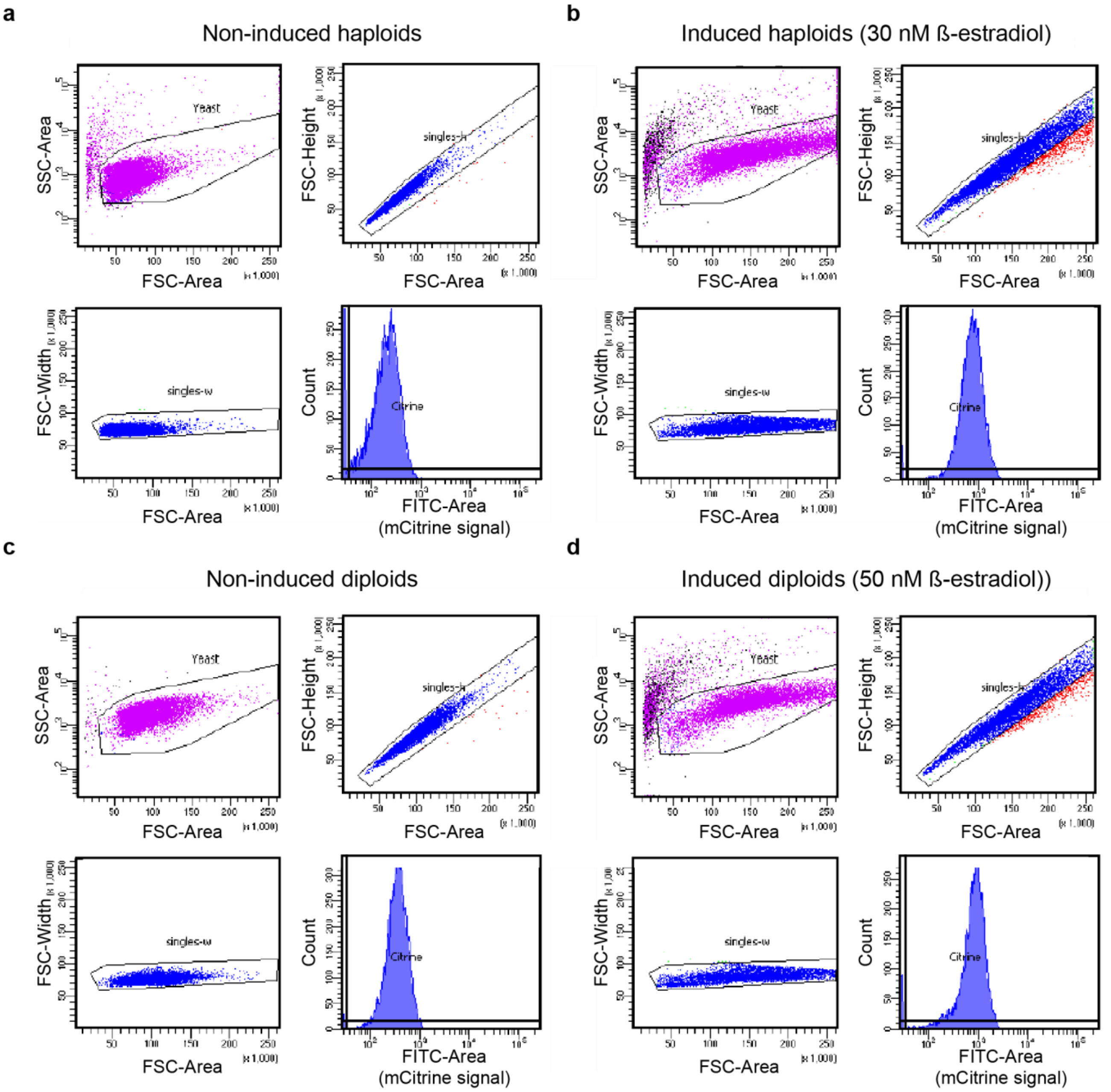

Supplementary Figure 11. Illustration of the gating strategy used during all the flow cytometry experiments, shown for representative yeast populations of non-induced haploids (a), induced haploids (b), non-induced diploids (c), and induced diploids (d). Panels show screen shots of the data acquired with FACSDiva 8.0.1 (BD) software during experiments. To differentiate the yeast population from background noise, the first gate (Yeast) was manually traced by comparing the side-scatter (SSC) area signal with the forward scatter (FSC) area signal. Potential doublets were then eliminated by comparing the height of the FSC signal to the area of the signal (manually traced gate: singles-h), and then comparing the width of the FSC signal to the area of the signal (manually traced gate: singles-w). At least 10.000 cells were then measured in the final gate and the mCitrine (FITC)

area signal was analyzed (Citrine). For each experiments and the corresponding replicates, identical gates were used.

Extended Data tables

Name	Genotype	Description	Origin	Fig.
ASY020-1	*Mat α/a; ADE2/ADE2, URA3/ura3, leu2/LEU2*	Non-inducible *WHI5*, diploid strain. Also used as microscopy background	Anika Seel, Schmoller lab (unpublished)	1, S1, S2
ASY023-1	*Mat α/a; ADE2/ADE2, URA3/ura3, WHI5/whi5Δ::kanMX6-LexAprom-WHI5-ADH1term-LEU2, his3/his3::LexA-ER-AD-TF-HIS3*	Inducible *WHI5*, diploid strain. Used as microscopy background	Anika Seel, Schmoller lab (unpublished)	4, 7, S7, S9
DBY001-2	*Mat a; ADE2, htb2Δ::KlacURA3*	Non-inducible *WHI5*, haploid *htb2Δ* strain	This study	S5
DBY002-1	*Mat a; ADE2, whi5Δ::kanMX6-LexAprom-WHI5-ADH1term-LEU2, his3::LexA-ER-AD-TF-HIS3, htb2Δ::KlacURA3*	Inducible *WHI5*, haploid *htb2Δ* strain	This study	Table S2
DBY003-1	*Mat a; ADE2, whi5Δ::kanMX6-LexAprom-WHI5-ADH1term-LEU2, his3::LexA-ER-AD-TF-HIS3, hho1Δ::CglaTRP1*	Inducible *WHI5*, haploid *hho1Δ* strain	This study	Table S2
DBY008-1	*Mat a; ADE2, whi5Δ::kanMX6-LexAprom-WHI5-ADH1term-LEU2, his3::LexA-ER-AD-TF-HIS3, hhf1Δ::CglaTRP1*	Inducible *WHI5*, haploid *hhf1Δ* strain	This study	Table S2
DBY009-1	*Mat α; ADE2, hhf2Δ::CglaTRP1*	Non-inducible *WHI5*, haploid *hhf2Δ* strain	This study	Table S2
DBY011-1	*Mat α; ADE2, hht1Δ::CglaTRP1*	Non-inducible *WHI5*, haploid *hht1Δ* strain	This study	Table S2
DBY013-1	*Mat α; ADE2, hht2Δ::CglaTRP1*	Non-inducible *WHI5*, haploid *hht2Δ* strain	This study	Table S2
DBY020-2	*Mat α; ADE2, ura3::CglaTRP1-HTB1prom-mCitrine-ADH1term-URA3*	Non-inducible *WHI5*, haploid strain with additional copy of *HTB1* promoter expressing *mCitrine*	This study	3, 6, S6, S8
DBY021-3	*Mat α; ADE2, ura3::CglaTRP1-HTB2prom-mCitrine-ADH1term-URA3*	Non-inducible *WHI5*, haploid strain with additional copy of *HTB2* promoter expressing *mCitrine*	This study	3, S6
DBY022-1	*Mat α; ADE2, ura3::CglaTRP1-HHF1prom-mCitrine-ADH1term-URA3*	Non-inducible *WHI5*, haploid strain with additional copy of *HHF1* promoter expressing *mCitrine*	This study	3, 6, S6, S8
DBY027-11	*Mat α; ADE2, ura3::CglaTRP1-HTB1prom-mCitrine-ADH1term-URA3, his3::ACT1prom-mKate2-ADH1term-HIS3, htb2Δ::LEU2*	Non-inducible *WHI5*, haploid *htb2Δ* strain with additional copy of *HTB1* promoter expressing *mCitrine* and additional copy of *ACT1* promoter expressing *mKate2*	This study	8
DCY003-6	*Mat α; ADE2, htb1::HTB1-linker-mCitrine-ADH1term-URA3*	Non-inducible *WHI5*, haploid strain with *HTB1* tagged with *mCitrine*	This study	1
DCY008-8	*Mat α; ADE2, ura3::CglaTRP1-HTB1prom-mCitrine-ADH1term-URA3, his3::ACT1prom-*	Non-inducible *WHI5*, haploid strain with	This study	8

Name	Genotype	Description	Origin	Fig.
	mKate2-ADH1term-HIS3	additional copy of *HTB1* promoter expressing *mCitrine* and additional copy of *ACT1* promoter expressing *mKate2*		
KCY001-3	*Mat a; ADE2, htb2::HTB2-linker-mCitrine-ADH1term-CglaTRP1, whi5Δ::kanMX6-LexAprom-WHI5-ADH1term-LEU2, his3::LexA-ER-AD-TF-HIS3*	Inducible *WHI5*, haploid strain with *HTB2* tagged with *mCitrine*	This study	1, S1, S2
KCY002-3	*Mat α; ADE2, htb2::HTB2-linker-mCitrine-ADH1term-CglaTRP1*	Non-inducible *WHI5*, haploid strain with *HTB2* tagged with *mCitrine*	This study	1, S1, S2
KCY005-1	*Mat α/a; ADE2/ADE2, whi5Δ::CglaTRP1/whi5Δ::kanMX6-LexAprom-WHI5-ADH1term-LEU2, his3/his3::LexA-ER-AD-TF-HIS3*	Inducible *WHI5*, diploid strain.<br>Also used as microscopy background	This study	1, 2, S1, S2, S5, S7
KCY006-1	*Mat α/a; ADE2/ADE2, htb2Δ::KlacURA3/HTB2, whi5Δ::CglaTRP1/whi5Δ::kanMX6-LexAprom-WHI5-ADH1term-LEU2, his3/his3::LexA-ER-AD-TF-HIS3*	Inducible *WHI5*, diploid strain with one *HTB2* allele deleted	This study	2, S5
KCY007-2	*Mat α; ADE2, ura3::CglaTRP1-150bpHHF1prom-mCitrine-ADH1term- URA3*	Non-inducible *WHI5*, haploid strain with additional 150 bp of *HHF1* promoter (truncated 5’ – 3’) expressing *mCitrine*	This study	6
KCY008-1	*Mat α; ADE2, ura3::CglaTRP1-300bpHHF1prom-mCitrine-ADH1term-URA3*	Non-inducible *WHI5*, haploid strain with additional 300 bp of *HHF1* promoter (truncated 5’ – 3’) expressing *mCitrine*	This study	6, S8
KCY009-1	*Mat α; ADE2, ura3::CglaTRP1-450bpHHF1prom-mCitrine-ADH1term-URA3*	Non-inducible *WHI5*, haploid strain with additional 450 bp of *HHF1* promoter (truncated 5’ – 3’) expressing *mCitrine*	This study	6, S8
KCY010-2	*Mat α; ADE2, ura3::CglaTRP1-600bpHHF1prom-mCitrine-ADH1term-URA3*	Non-inducible *WHI5*, haploid strain with additional 600 bp of *HHF1* promoter (truncated 5’ – 3’) expressing *mCitrine*	This study	6
KCY011-1	*Mat a; ADE2, ura3::CglaTRP1-150bpHHF1prom-mCitrine-ADH1term-URA3, whi5Δ::kanMX6-LexAprom-WHI5-ADH1term-LEU2, his3::LexA-ER-AD-TF-HIS3*	Inducible *WHI5*, haploid strain with additional 150 bp of *HHF1* promoter (truncated 5’ – 3’) expressing *mCitrine*	This study	6
KCY012-1	*Mat a; ADE2, ura3::CglaTRP1-300bpHHF1prom-mCitrine-ADH1term-URA3, whi5Δ::kanMX6-LexAprom-WHI5-ADH1term-LEU2, his3::LexA-ER-AD-TF-HIS3*	Inducible *WHI5*, haploid strain with additional 300 bp of *HHF1* promoter (truncated 5’ – 3’)	This study	6, S8

Name	Genotype	Description	Origin	Fig.
		expressing *mCitrine*		
KCY013-1	*Mat a; ADE2, ura3::CglaTRP1-450bpHHF1prom-mCitrine-ADH1term-URA3, whi5Δ::kanMX6-LexAprom-WHI5-ADH1term-LEU2, his3::LexA-ER-AD-TF-HIS3*	Inducible *WHI5*, haploid strain with additional 450 bp of *HHF1* promoter (truncated 5' – 3') expressing *mCitrine*	This study	6, S8
KCY014-1	*Mat a; ADE2, ura3::CglaTRP1-600bpHHF1prom-mCitrine-ADH1term-URA3, whi5Δ::kanMX6-LexAprom-WHI5-ADH1term-LEU2, his3::LexA-ER-AD-TF-HIS3*	Inducible *WHI5*, haploid strain with additional 600 bp of *HHF1* promoter (truncated 5' – 3') expressing *mCitrine*	This study	6
KCY015-1	*Mat a; ADE2, ura3::CglaTRP1-150bpHTB1prom-mCitrine-ADH1term-URA3, whi5Δ::kanMX6-LexAprom-WHI5-ADH1term-LEU2, his3::LexA-ER-AD-TF-HIS3*	Inducible *WHI5*, haploid strain with additional 150 bp of *HTB1* promoter (truncated 5' – 3') expressing *mCitrine*	This study	6
KCY016-1	*Mat a; ADE2, ura3::CglaTRP1-300bpHTB1prom-mCitrine-ADH1term-URA3, whi5Δ::kanMX6-LexAprom-WHI5-ADH1term-LEU2, his3::LexA-ER-AD-TF-HIS3*	Inducible *WHI5*, haploid strain with additional 300 bp of *HTB1* promoter (truncated 5' – 3') expressing *mCitrine*	This study	6, S8
KCY017-1	*Mat a; ADE2, ura3::CglaTRP1-450bpHTB1prom-mCitrine-ADH1term-URA3, whi5Δ::kanMX6-LexAprom-WHI5-ADH1term-LEU2, his3::LexA-ER-AD-TF-HIS3*	Inducible *WHI5*, haploid strain with additional 450 bp of *HTB1* promoter (truncated 5' – 3') expressing *mCitrine*	This study	6, S8
KCY018-1	*Mat a; ADE2, ura3::CglaTRP1-600bpHTB1prom-mCitrine-ADH1term-URA3, whi5Δ::kanMX6-LexAprom-WHI5-ADH1term-LEU2, his3::LexA-ER-AD-TF-HIS3*	Inducible *WHI5*, haploid strain with additional 600 bp of *HTB1* promoter (truncated 5' – 3') expressing *mCitrine*	This study	6
KCY019-1	*Mat a; ADE2, ura3::CglaTRP1-750bpHTB1prom-mCitrine-ADH1term-URA3, whi5Δ::kanMX6-LexAprom-WHI5-ADH1term-LEU2, his3::LexA-ER-AD-TF-HIS3*	Inducible *WHI5*, haploid strain with additional 750 bp of *HTB1* promoter (truncated 5' – 3') expressing *mCitrine*	This study	6
KCY020-1	*Mat α; ADE2, ura3::CglaTRP1-150bpHTB1prom-mCitrine-ADH1term-URA3*	Non-inducible *WHI5*, haploid strain with additional 150 bp of *HTB1* promoter (truncated 5' – 3') expressing *mCitrine*	This study	6
KCY021-1	*Mat α; ADE2, ura3::CglaTRP1-300bpHTB1prom-mCitrine-ADH1term-URA3*	Non-inducible *WHI5*, haploid strain with additional 300 bp of *HTB1* promoter (truncated 5' – 3') expressing *mCitrine*	This study	6, S8
KCY022-1	*Mat α; ADE2, ura3::CglaTRP1-450bpHTB1prom-mCitrine-ADH1term-URA3*	Non-inducible *WHI5*, haploid strain with additional 450 bp of	This study	6, S8

Name	Genotype	Description	Origin	Fig.
		HTB1 promoter (truncated 5' – 3') expressing *mCitrine*		
KCY023-4	*Mat α; ADE2, ura3::CglaTRP1-600bpHTB1prom-mCitrine-ADHterm-URA3*	Non-inducible *WHI5*, haploid strain with additional 600 bp of *HTB1* promoter (truncated 5' – 3') expressing *mCitrine*	This study	6
KCY024-1	*Mat α; ADE2, ura3::CglaTRP1-750bpHTB1prom-mCitrine-ADH1term-URA3*	Non-inducible *WHI5*, haploid strain with additional 750 bp of *HTB1* promoter (truncated 5' – 3') expressing *mCitrine*	This study	6
KCY027-4	*Mat α/a; ADE2/ADE2, htb2::HTB2-linker-mCitrine-ADH1term-CglaTRP1 /htb2::HTB2-linker-mCitrine-ADH1term-KlacURA3*	Non-inducible *WHI5*, diploid strain with both *HTB2* alleles tagged with *mCitrine*	This study	1, S1, S2
KCY028-1	*Mat α/a; ADE2/ADE2, htb2::HTB2-linker-mCitrine-ADH1term-KlacURA3/htb2::HTB2-linker-mCitrine-ADH1term-CglaTRP1, whi5Δ::CglaTRP1/whi5Δ::kanMX6-LexAprom-WHI5-ADH1term-LEU2, his3/his3::LexA-ER-AD-TF-HIS3*	Inducible *WHI5*, diploid strain with both *HTB2* alleles tagged with *mCitrine*	This study	1, S1, S2
KCY029-1	*Mat α/a; ADE2/ADE2, htb2Δ::KlacURA3/htb2::HTB2-linker-mCitrine-ADH1term-CglaTRP1, whi5Δ::CglaTRP1/whi5Δ::kanMX6-LexAprom-WHI5-ADH1term-LEU2, his3/his3::LexA-ER-AD-TF-HIS3*	Inducible *WHI5*, diploid strain with one *HTB2* allele deleted and the other *HTB2* allele tagged with *mCitrine*	This study	1, S1, S2
KCY031-1	*Mat α/a; ADE2/ADE2, ura3::CglaTRP1-HTB1prom-mCitrine-ADH1term-URA3/ura3, WHI5/whi5Δ::kanMX6-LexAprom-WHI5-ADH1term-LEU2, his3/his3::LexA-ER-AD-TF-HIS3*	Inducible *WHI5*, diploid strain with additional copy of *HTB1* promoter expressing *mCitrine*	This study	3, 4, 6, 7, S6, S7, S8
KCY032-2	*Mat α/a; ADE2/ADE2, ura3::CglaTRP1-HHF1prom-mCitrine-ADH1term-URA3/ura3, WHI5/whi5Δ::kanMX6-LexAprom-WHI5-ADH1term-LEU2, his3/his3::LexA-ER-AD-TF-HIS3*	Inducible *WHI5*, diploid strain with additional copy of *HHF1* promoter expressing *mCitrine*	This study	3, 4, 6, 7, S6, S7, S8
KCY033-2	*Mat α/a; ADE2/ADE2, ura3::CglaTRP1-HTB2prom-mCitrine-ADH1term-URA3/ura3, WHI5/whi5Δ::kanMX6-LexAprom-WHI5-ADH1term-LEU2, his3/his3::LexA-ER-AD-TF-HIS3*	Inducible *WHI5*, diploid strain with additional copy of *HTB2* promoter expressing *mCitrine*	This study	3, 4, S7, S8
KCY035-3	*Mat α/a; ADE2/ADE2, ura3::CglaTRP1-ACT1prom-mCitrine-ADH1term-URA3/ura3, WHI5/whi5Δ::kanMX6-LexAprom-WHI5-ADH1term-LEU2, his3/his3::LexA-ER-AD-TF-HIS3*	Inducible *WHI5*, diploid strain with additional copy of *ACT1* promoter expressing *mCitrine*	This study	3, 4, S7, S8
KCY038-1	*Mat α/a; ADE2/ADE2, ura3::CglaTRP1-300bpHTB1prom-mCitrine-ADH1term-URA3/ura3, leu2/LEU2*	Non-inducible *WHI5*, diploid strain with additional 300 bp of *HTB1* promoter (truncated 5' – 3') expressing *mCitrine*	This study	6, S8
KCY039-1	*Mat α/a; ADE2/ADE2, ura3::CglaTRP1-*	Inducible *WHI5*, diploid	This study	6, 7, S8,

Name	Genotype	Description	Origin	Fig.
	300bpHTB1prom-mCitrine-ADH1term-URA3/ura3, WHI5/whi5Δ::kanMX6-LexAprom-WHI5-ADH1term-LEU2, his3/his3::LexA-ER-AD-TF-HIS3	strain with additional 300 bp of *HTB1* promoter (truncated 5' – 3') expressing *mCitrine*		S9
KCY040-1	*Mat α/a; ADE2/ADE2, ura3::CglaTRP1-450bpHTB1prom-mCitrine-ADH1term-URA3/ura3, leu2/LEU2*	Non-inducible *WHI5*, diploid strain with additional 450 bp of *HTB1* promoter (truncated 5' – 3') expressing *mCitrine*	This study	6, S8
KCY041-1	*Mat α/a; ADE2/ADE2, ura3::CglaTRP1-450bpHTB1prom-mCitrine-ADH1term-URA3/ura3, WHI5/whi5Δ::kanMX6-LexAprom-WHI5-ADH1term-LEU2, his3/his3::LexA-ER-AD-TF-HIS3*	Inducible *WHI5*, diploid strain with additional 450 bp of *HTB1* promoter (truncated 5' – 3') expressing *mCitrine*	This study	6, 7, S8, S9
KCY043-1	*Mat α/a; ADE2/ADE2, ura3::CglaTRP1-300bpHHF1prom-mCitrine-ADH1term-URA3ura3, WHI5/whi5Δ::kanMX6-LexAprom-WHI5-ADH1term-LEU2, his3/his3::LexA-ER-AD-TF-HIS3*	Inducible *WHI5*, diploid strain with additional 300 bp of *HHF1* promoter (truncated 5' – 3') expressing *mCitrine*	This study	6, 7, S8, S9
KCY045-1	*Mat α/a; ADE2/ADE2, ura3::CglaTRP1-450bpHHF1prom-mCitrine-ADH1term-URA3/ura3, WHI5/whi5Δ::kanMX6-LexAprom-WHI5-ADH1term-LEU2, his3/his3::LexA-ER-AD-TF-HIS3*	Inducible *WHI5*, diploid strain with additional 450 bp of *HHF1* promoter (truncated 5' – 3') expressing *mCitrine*	This study	6, 7, S8, S9
KCY049-1	*Mat a; ADE2, ura3::CglaTRP1-HTB1prom-mCitrine-ADH1term-URA3, hir1Δ::natMX6, whi5Δ::kanMX6-LexAprom-WHI5-ADH1term-LEU2, his3::LexA-ER-AD-TF-HIS3*	Inducible *WHI5*, haploid *hir1Δ* strain with additional copy of *HTB1* promoter expressing *mCitrine*	This study	S10
KCY050-2	*Mat α/a; ADE2/ADE2, htb1::HTB1-linker-mCitrine-ADH1term-KlacURA3/htb1::HTB1-linker-mCitrine-ADH1term-CglaTRP1*	Non-inducible *WHI5*, diploid strain with both *HTB1* alleles tagged with *mCitrine*	This study	1
KCY051-1	*Mat a; ADE2, ura3::CglaTRP1-HTB2prom-mCitrine-ADH1term-URA3, hir1Δ::natMX6, whi5Δ::kanMX6-LexAprom-WHI5-ADH1term-LEU2, his3::LexA-ER-AD-TF-HIS3*	Inducible *WHI5*, haploid *hir1Δ* strain with additional copy of *HTB2* promoter expressing *mCitrine*	This study	S10
KSY212-2	*Mat α; ADE2, rrp6Δ::CglaTRP1*	Non-inducible *WHI5*, haploid *rrp6Δ* strain	This study	2, S5
KSY213-6	*Mat a; ADE2, rrp6Δ::CglaTRP1, whi5Δ::kanMX6-LexAprom-WHI5-ADH1term-LEU2, his3::LexA-ER-AD-TF-HIS3*	Inducible *WHI5*, haploid *rrp6Δ* strain	This study	2, S5
KSY214-1	*Mat α; ADE2, hir1Δ::CglaTRP1*	Non-inducible *WHI5*, haploid *hir1Δ* strain	This study	2, S5
KSY215-2	*Mat a; ADE2, hir1Δ::CglaTRP1, whi5Δ::kanMX6-LexAprom-WHI5-ADH1term-LEU2, his3::LexA-ER-AD-TF-HIS3*	Inducible *WHI5*, haploid *hir1Δ* strain	This study	2, S5
KSY219-3	*Mat a; ADE2, rtt106Δ::CglaTRP1, whi5Δ::kanMX6-LexAprom-WHI5-ADH1term-LEU2, his3::LexA-ER-AD-TF-HIS3*	Inducible *WHI5*, haploid *rtt106Δ* strain	This study	2, S5
KSY208-3	*Mat α; ADE2, ura3::mCitrine-ADH1term-URA3*	Non-inducible *WHI5*, haploid strain with	This study	

Name	Genotype	Description	Origin	Fig.
		additional *mCitrine* copy (not expressed)		
KSY222-1	*Mat a; ADE2, ura3::CglaTRP1-HTB1prom-mCitrine-ADH1term-URA3, whi5Δ::kanMX6-LexAprom-WHI5-ADH1term-LEU2, his3::LexA-ER-AD-TF-HIS3*	Inducible *WHI5*, haploid strain with additional copy of *HTB1* promoter expressing *mCitrine*	This study	3, 4, S6, S10
KSY223-3	*Mat a; ADE2, ura3::CglaTRP1-HHF1prom-mCitrine-ADH1term-URA3, whi5Δ::kanMX6-LexAprom-WHI5-ADH1term-LEU2, his3::LexA-ER-AD-TF-HIS3*	Inducible *WHI5*, haploid strain with additional copy of *HHF1* promoter expressing *mCitrine*	This study	3, 4, S6
KSY225-2	*Mat a; ADE2, ura3::CglaTRP1-HTB2prom-mCitrine-ADH1term-URA3, whi5Δ::kanMX6-LexAprom-WHI5-ADH1term-LEU2, his3::LexA-ER-AD-TF-HIS3*	Inducible *WHI5*, haploid strain with additional copy of *HTB2* promoter expressing *mCitrine*	This study	3, 4, S6, S10
KSY226-3	*Mat a; ADE2, ura3::mCitrine-ADH1term-URA3, whi5Δ::kanMX6-LexAprom-WHI5-ADH1term-LEU2, his3::LexA-ER-AD-TF-HIS3*	Inducible *WHI5*, haploid strain with additional *mCitrine* copy (not expressed)	This study	
KSY229-1	*Mat α; ADE2, ura3::CglaTRP1-ACT1prom-mCitrine-ADH1term-URA3*	Non-inducible *WHI5*, haploid strain with additional copy of *ACT1* promoter expressing *mCitrine*	This study	3, S6
KSY230-1	*Mat a; ADE2, ura3::CglaTRP1-ACT1prom-mCitrine-ADH1term-URA3, whi5Δ::kanMX6-LexAprom-WHI5-ADH1term-LEU2, his3::LexA-ER-AD-TF-HIS3*	Inducible *WHI5*, haploid strain with additional copy of *ACT1* promoter expressing *mCitrine*	This study	3, S6
MMY116-2C	*Mat α; ADE2*	Non-inducible *WHI5*, haploid strain. Also used as microscopy background	Skotheim lab stock	1, 2, 3, S1, S2, S3, S4, S5
MS62-1	*Mat a; ADE2, whi5Δ::kanMX6, his3::LexA-ER-AD-TF-HIS3*	β-estradiol dependent transcription factor, haploid *whi5Δ* strain	Matthew Swaffer, Skotheim lab	S3
MS63-1	*Mat a; ADE2, whi5Δ::kanMX6-LexAprom-WHI5-ADH1term-LEU2, his3::LexA-ER-AD-TF-HIS3*	Inducible *WHI5*, haploid strain. Also used as microscopy background	Matthew Swaffer, Skotheim lab	1, 2, 3, S1, S2, S3, S4, S5

Supplementary Table 1. Yeast strains used in this work. All strains are based on W303. *CglaTRP1* denotes the *TRP1* gene of the organism *C. glabrata*, *KlacURA3* denotes the *URA3* gene of the organism *K. lactis*.

qPCR primer	MS63-1 [mean C_p^{Gene}]	Δ strain [Cp range]
HHO1	20.3 ± 0.1	$no\ amp$
HTB2	16.8 ± 0.1	$40.9 - no\ amp$
HHF1	17.7 ± 0.3	$34.6\ -\ no\ amp$
HHF2	19.5 ± 0.1	$33.6 - 35.9$
HHT1	18.0 ± 0.1	$32.9 - no\ amp$
HHT2	19.0 ± 1.4	$33.7 - no\ amp$

Supplementary Table 2. Results of qPCR measurements on deletion strains to test for primer specificity. C_p^{Gene} for the MS63-1 strain are the mean of n = 3 technical replicates, with standard deviation of the mean. For the deletion strains, C_p-ranges reach from the minimum C_p^{min} to maximum C_p^{max} values of n = 3 technical replicates for *HHO1*, *HTB2*, *HHT2* and n = 6 technical replicates for *HHF1*, *HHF2*, *HHT1*. No detectable amplification curve over threshold is denoted as "$no\ amp$".

Gene	qPCR primer direction	qPCR primer sequence (5’ - 3’)
ACT1	forward	AGTTGCCCCAGAAGAACACC
	reverse	GGACAAAACGGCTTGGATGG
ENO2	forward	TTGTTCCATCTGGTGCCTCC
	reverse	ACGAAAGCAGCAGCAATGAC
HHF1	forward	TACACCGAACACGCCAAGAG
	reverse	TTGCTTGTTGTTACCGTTTTCTT
HHF2	forward	ACGAAGAAGTCAGAGCCGTC
	reverse	ACCGATTGTTTAACCACCGATTG
HHO1	forward	ACCAGCAAAGGCAAGGAGAA
	reverse	AAAGCCGTGAGCCCTTCAAT
HHT1	forward	CAATCTTCTGCCATCGGTGC
	reverse	ACTGATGACAATCAACAAACTATGA
HHT2	forward	AGCAAACACTCCACAATGGC
	reverse	CAAGGCAACAGTACCTGGCT
HTA1	forward	GTTGCCAAAGAAGTCTGCCA
	reverse	CAGTTTAGTTCCTTCCGCCTT
HTA2	forward	TCGCCCAAGGTGGTGTTTT
	reverse	TGATTTGCTTTGTTTCTTTTCAACT
HTB1	forward	TACACACATACAATGTCTGCTAAAG
	reverse	AGTGTCAGGGTGAGTTTGCTT
HTB2	forward	CCTCTGCCGCCGAAAAGAAA
	reverse	TCTTACCATCGACGGAGGTTG
mCitrine	forward	GAGCTGAAGGGCATCGACTT
	reverse	TTCTGCTTGTCGGCCATGAT
RDN18	forward	AACTCACCAGGTCCAGACACAATAAGG
	reverse	AAGGTCTCGTTCGTTATCGCAATTAAGC
RPB1	forward	CCAGAAGTGGTCACACCATATAA
	reverse	GGTCTCCGCTATCACGAATG
RPB3	forward	TGTGGGGTCTATTCCCGTTG
	reverse	CGCCCGTCATCATTACGTCT

Supplementary Table 3. Sequences of qPCR primers used in this work.
