# Supplementary material for: Transcription coordinates histone amounts and genome content
Source: Nat Commun. 2021 Jul 9;12:4202. doi: 10.1038/s41467-021-24451-8 (PMC8270936; doi:10.1038/s41467-021-24451-8)
Supplement: Supplementary file 2 — Reporting Summary [file 41467_2021_24451_MOESM2_ESM.pdf]

nature research

Corresponding author(s): Kurt Schmoller

Last updated by author(s): Jun 16, 2021

Reporting Summary

Nature Research wishes to improve the reproducibility of the work that we publish. This form provides structure for consistency and transparency in reporting. For further information on Nature Research policies, see our Editorial Policies and the Editorial Policy Checklist.

Statistics

For all statistical analyses, confirm that the following items are present in the figure legend, table legend, main text, or Methods section.

n/a	Confirmed	
☐	☒	The exact sample size (*n*) for each experimental group/condition, given as a discrete number and unit of measurement
☒	☐	A statement on whether measurements were taken from distinct samples or whether the same sample was measured repeatedly
☐	☒	The statistical test(s) used AND whether they are one- or two-sided<br>*Only common tests should be described solely by name; describe more complex techniques in the Methods section.*
☒	☐	A description of all covariates tested
☐	☒	A description of any assumptions or corrections, such as tests of normality and adjustment for multiple comparisons
☐	☒	A full description of the statistical parameters including central tendency (e.g. means) or other basic estimates (e.g. regression coefficient) AND variation (e.g. standard deviation) or associated estimates of uncertainty (e.g. confidence intervals)
☐	☒	For null hypothesis testing, the test statistic (e.g. *F*, *t*, *r*) with confidence intervals, effect sizes, degrees of freedom and *P* value noted<br>*Give P values as exact values whenever suitable.*
☒	☐	For Bayesian analysis, information on the choice of priors and Markov chain Monte Carlo settings
☒	☐	For hierarchical and complex designs, identification of the appropriate level for tests and full reporting of outcomes
☒	☐	Estimates of effect sizes (e.g. Cohen's *d*, Pearson's *r*), indicating how they were calculated

Our web collection on statistics for biologists contains articles on many of the points above.

Software and code

Policy information about availability of computer code

Data collection	Zeiss (Zen 2.3 (blue edition)) microscope software, BD FACSDiva 8.0.1 flow cytometry software
Data analysis	Fiji (ImageJ 1.52e), Matlab R2017b, FISHquant v.3

For manuscripts utilizing custom algorithms or software that are central to the research but not yet described in published literature, software must be made available to editors and reviewers. We strongly encourage code deposition in a community repository (e.g. GitHub). See the Nature Research guidelines for submitting code & software for further information.

Data

Policy information about availability of data

All manuscripts must include a data availability statement. This statement should provide the following information, where applicable:

- Accession codes, unique identifiers, or web links for publicly available datasets
- A list of figures that have associated raw data
- A description of any restrictions on data availability

Yeast strains and raw data are available upon reasonable request.

Field-specific reporting

Life sciences study design

All studies must disclose on these points even when the disclosure is negative.

Sample size	Microscopy and smFISH experiments are based on at least two independent biological replicates. qPCR measurements are based on at least 6 independent biological replicates, except for the deletion experiments shown in Fig. 2f-h and Fig. S5c-e, which are based on 2 to 12 replicates per condition, and the control experiments shown in Fig. S3b & S3c, which are based on at least 4 replicates per condition. Flow cytometry measurements are based on at least 4 independent biological replicates. No sample size calculation was performed. For each type of measurement, replicate numbers were chosen such that major phenotypes could be robustly detected. Replicate number per day was in most cases limited by the number of samples that could be processed simultaneously.
Data exclusions	No data were excluded.
Replication	Each experiment was performed at least twice. Before pooling data, we always compared results from different experiments. No qualitative differences were noted.
Randomization	Strains and conditions that were directly compared were typically measured in parallel in a single experiment, making further randomization not applicable.
Blinding	To avoid biases, bud counts were performed by different people using blinded samples. Analysis of qPCR and flow cytometry did not involve manual data selection, and mage analysis was automated as far as possible to avoid potential biases.

Reporting for specific materials, systems and methods

We require information from authors about some types of materials, experimental systems and methods used in many studies. Here, indicate whether each material, system or method listed is relevant to your study. If you are not sure if a list item applies to your research, read the appropriate section before selecting a response.

Materials & experimental systems

n/a	Involved in the study
☒	☐ Antibodies
☒	☐ Eukaryotic cell lines
☒	☐ Palaeontology and archaeology
☒	☐ Animals and other organisms
☒	☐ Human research participants
☒	☐ Clinical data
☒	☐ Dual use research of concern

Methods

n/a	Involved in the study
☒	☐ ChIP-seq
☐	☒ Flow cytometry
☒	☐ MRI-based neuroimaging

Flow Cytometry

Plots

Confirm that:

☒ The axis labels state the marker and fluorochrome used (e.g. CD4-FITC).

☒ The axis scales are clearly visible. Include numbers along axes only for bottom left plot of group (a 'group' is an analysis of identical markers).

☐ All plots are contour plots with outliers or pseudocolor plots.

☒ A numerical value for number of cells or percentage (with statistics) is provided.

Methodology

Sample preparation	Log-phase yeast cultures were grown in liquid media and harvested below OD=1.3. Cells were fixed using a 37% formaldehyde solution. For cell cycle analysis, cells were fixed with 80% cold ethanol and DNA was stained using 10x SYBR Green 1 for 1 hour.
Instrument	BD Biosciences, LSR II
Software	BD FACSDiva 8.0.1 Software
Cell population abundance	At least 10000 cells in the final gate were measured for each sample.

Gating strategy	To gate cells, we first removed obvious outliers in the SSC-A vs FSC-A plot, then in the FSC-H vs FSC-A plot, finally in the FSC-W vs FSC-A plot. We always ensured that the gating included at least 90% of all counted objects.

☒ Tick this box to confirm that a figure exemplifying the gating strategy is provided in the Supplementary Information.
